# Exploratory baseline mucosal transcriptomics and vedolizumab response in ulcerative colitis

**DOI:** 10.1371/journal.pone.0361301

**Published:** 2026-10-26

**Authors:** Liang Zhang, Jian Liu, Chenfei Liang, Qiang Han, Da Li, Yali Wang

**Affiliations:** Department of Gastroenterology, The First Hospital of Tsinghua University, School of Clinical Medicine, Tsinghua Medicine, Tsinghua University, Beijing, China; Charite Universitatsmedizin Berlin, GERMANY

## Abstract

Currently, there are still no reliable pre-treatment biomarkers for vedolizumab in ulcerative colitis. To investigate baseline mucosal signals, we reconstructed the discovery cohort from GSE73661 and evaluated the derived panel in the blood samples from GSE191328 and the independent mucosal RNA-seq cohorts from E-MTAB-7845. The reconstructed cohort comprised 24 baseline samples, including 14 composite responders and 10 non-responders. Through repeated LASSO variable selection, the study identified an exploratory gene set consisting of *ZNF689*, *TPRXL*, *ZC3H10*, and *MRPL55*. The selection procedure yielded a mean held-out AUC of 0.611 across 18 complete nested-validation repetitions; this does not independently validate the final gene set. When the fixed gene set was projected exploratorily, the AUC for 17 blood samples was 0.458 (95% CI 0.162–0.755), and the AUC for 27 independent UC mucosal samples was 0.534 (95% CI 0.293–0.775). The apparent AUC of this gene set on the discovery set was 1.000, and 500 full-process bootstrap analyses indicated that gene selection was unstable. Among the three targeted inflammatory panels, interferon-γ showed a directional difference that was not statistically significant (mean GSVA score difference −0.425; nominal *P* = 0.066; adjusted *P* = 0.197). Expression scales and clinical endpoints differed across cohorts. The above results describe post-selection separation within the discovery cohort and do not establish reproducible predictive performance, tissue specificity, or a definitive four-gene signature.

## Introduction

Although the α4β7 integrin antagonist vedolizumab is an established treatment for ulcerative colitis, it is still associated with high rates of incomplete endoscopic healing and primary nonresponse [1]. Therefore, before initiating treatment, clinicians need tools that can link the drug’s mechanism of action to individual inflammatory characteristics, rather than relying solely on descriptive tissue signatures. The gut-selective mechanism provides a rationale for examining baseline intestinal mucosa for candidate response markers.

Vedolizumab modulates the gut trafficking axis, promoting both changes in the mucosal transcriptome and histological healing [2]. Meanwhile, baseline mucosal profiling by Verstockt and Gazouli suggests that pre-treatment expression patterns in the colon are associated with the ultimate efficacy of vedolizumab [3,4]. Although recent blood-based studies show promise [5], whole-blood biomarkers may not fully reflect the local tissue dynamics essential to this drug’s mechanism of action [6].

Baseline mucosal profiling also fits the precision-medicine aim of integrating clinical, endoscopic, and molecular data, without replacing clinical assessment with unproven scores [7].

Recent translational studies have described local processes associated with vedolizumab response, including communication between TH17 cells and monocytes, α4β7-dependent adhesion, CD4 + cell infiltration, and changes in the state of the stroma or mononuclear phagocytes. In conjunction with the remodeling of gut-associated lymphoid tissue, these findings suggest a response pattern that differs in part from that of anti-TNF therapy [8–15]. These observations motivate testing baseline mucosal markers for reproducible discrimination and biological interpretability. Fig 1 summarizes the overall study design and analytical workflow.

**Fig 1:**
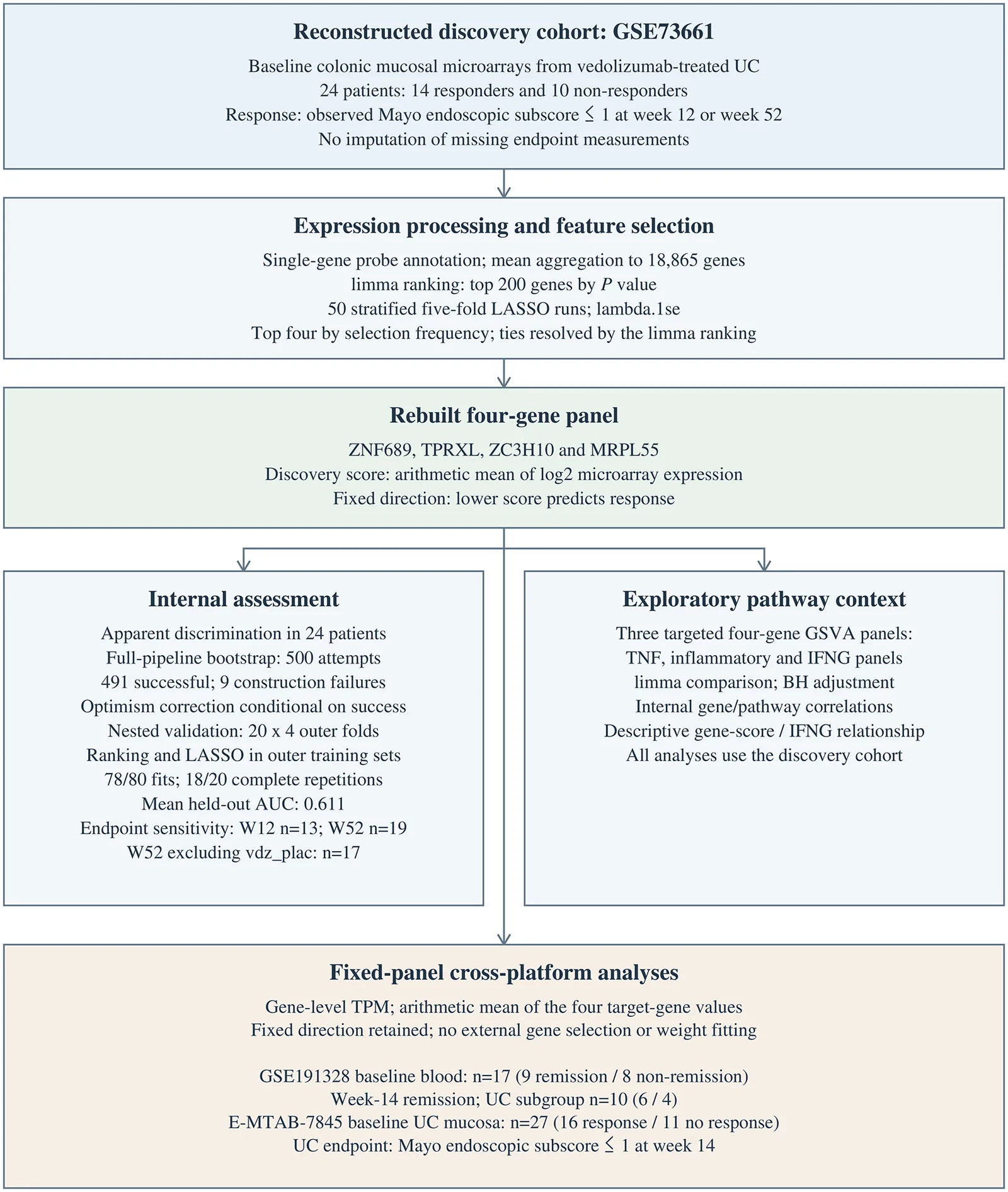
Study design and reconstructed analysis workflow. In this study, 24 baseline UC mucosal samples (14 composite responders and 10 non-responders) were obtained from GSE73661. After internal evaluation of the reconstructed panel, the panel was projected exploratorily onto 17 blood samples from GSE191328 (9 in remission/8 not in remission) and 27 UC mucosal samples from E-MTAB-7845 (16/11). Differences among cohorts involved clinical endpoint, expression scales, and baseline population features. Bootstrap correction was contingent upon successful panel construction. Repeated nested validation was performed by repeating label-based selection within each outer training subset.

Based on publicly available transcriptomic datasets, this study evaluated the feasibility of using baseline mucosal gene expression to distinguish responders from non-responders to vedolizumab. To test whether the derived gene panel possesses cross-cohort generalizability, we further evaluated it in independent blood and mucosal cohorts. This is a discovery-phase study and is not intended to establish a clinical predictive model. The baseline characteristics of each cohort are shown in Table 1.

**Table 1:** Analysis cohorts and contextual public resources.

Category	Resource	Tissue / domain	Platform / material	Scope	Role
Discovery	GSE73661	Colonic mucosa	Affymetrix HuGene 1.0 ST	24 UC (14 R/10 NR)	Reconstructed baseline cohort; composite W12/W52 outcome
External projection	GSE191328	Peripheral blood	RNA-seq TPM	17 IBD (9/8); UC subset 10 (6/4)	Exploratory cross-platform transfer; W14 remission
External projection	E-MTAB-7845	Colonic mucosa	RNA-seq TPM; GENCODE v47	27 UC (16/11); Inception 20; Validation 7	Exploratory independent same-tissue projection; W14 Mayo endoscopic subscore ≤1
Endpoint sensitivity	GSE73661	Colonic mucosa	Fixed rebuilt panel	W12: 13 (3/10); W52: 19 (13/6)	Observed timepoint outcomes; overlapping discovery subsets
Sample audit	GSE73661	Baseline Mayo endoscopic subscore	Public metadata and saved matrix	24 baseline samples; endoscopic subscore 2–3	Retention and missingness documented in S2 Table

## Results

### Discovery cohort composition and baseline transcriptomic landscape

The reconstructed discovery set comprised 24 baseline mucosal biopsies from patients with UC treated with vedolizumab (14 responders/10 non-responders). Substantial interindividual variability was evident on principal component analysis (PCA; Fig 2A) and hierarchical clustering (Fig 2B). In the exploratory PCA shown in Fig 2A, the sample from patient 64 deviated visually. However, since the available processed data and metadata did not establish a technical reason for exclusion, the entire set of 24 samples was retained (S2 Table). The recovered records indicate a pooled primary analysis of 24 samples and a separate sensitivity analysis of 23 samples excluding patient 64 (14 responders/9 non-responders). No predefined technical exclusion criteria were found; separation based solely on PCA does not justify the exclusion of this sample. The submitted 23-sample model estimates could not be matched to complete execution records among the recovered materials (S5 Data).

**Fig 2:**
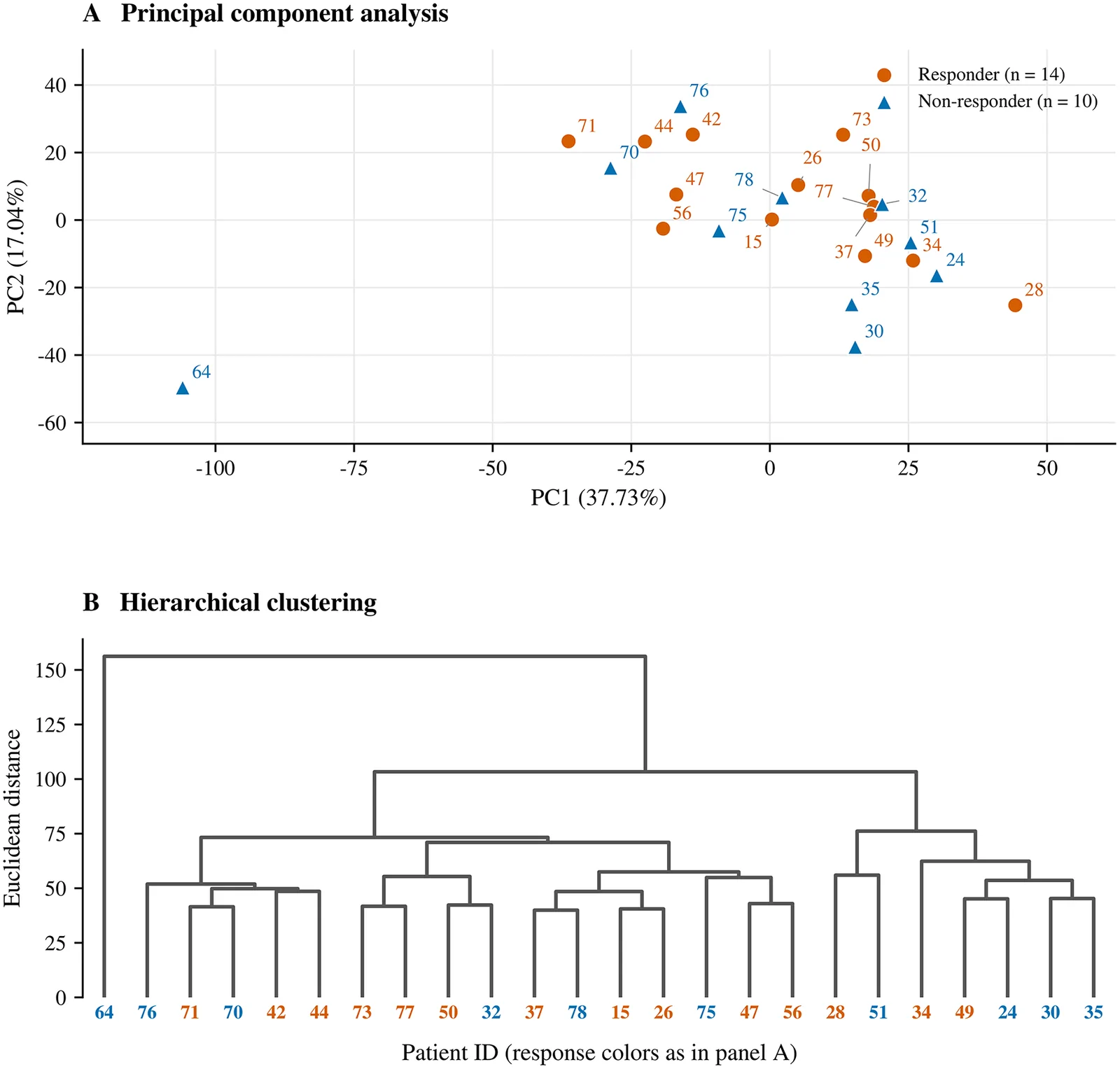
Exploratory map of the reconstructed discovery cohort. (A) Principal component analysis (PCA) of centered, unscaled expression levels for 18,865 genes across 24 baseline samples (14 responders/10 non-responders); PC1 and PC2 explain 37.73% and 17.04% of the variance, respectively. (B) Euclidean distance-based complete-linkage hierarchical clustering. Patient labels are consistent with S2 Table. Samples are from GSE73661. Patient 64 was retained; labels map to GSM accessions in S2 Table.

None of the response-associated candidate transcripts identified by limma at the single-gene level passed false discovery rate (FDR) correction. Therefore, we do not consider individual genes to be independent discoveries, but rather view them purely as a ranking framework for constructing a compact exploratory panel.

### A rebuilt four-gene panel shows in-sample separation with unstable composition

Following repeated LASSO variable selection, a four-gene mucosal panel consisting of *ZNF689*, *TPRXL*, *ZC3H10*, and *MRPL55* was identified. Due to changes in probe filtering and the selection process during the reconstruction, this panel differs from the previously submitted panel containing *TVP23C*. Across 50 runs, these genes were selected 41, 38, 36, and 20 times, respectively. Given that the selection frequencies of *MRPL55* and *SIRPD* were tied, the tie was resolved using limma rank (23rd versus 55th). As a fixed panel, its score showed complete apparent separation in the discovery cohort (Fig 3B–C; AUC 1.000). Given that the model’s variable selection and performance evaluation shared the same observational data, this result represents an in-sample fit and is not an unbiased estimate of predictive performance. The two probes associated with *TVP23C* were filtered out because they mapped to multiple gene symbols, thereby violating the single-gene annotation rule (S5 Table).

When the entire derivation process was rerun using bootstrap resampling, both model performance and stability decreased. We successfully built four-gene panels in 491 of 500 attempts (9 failed to reach four genes). Successful constructions had an average optimism of 0.075 and an optimism-corrected AUC of 0.925. The average AUCs for the bootstrap training set and the original cohort evaluation were 0.808 and 0.733, respectively. A total of 92 training set AUCs and 82 original cohort AUCs were below 0.5; the study did not artificially inflate these values by reversing the prediction direction. Successful panels included a total of 610 distinct genes, and MRPL55 recurred 30 times out of 491 attempts. Fig 3D–3E summarizes optimism and selection instability. Independent R refits reproduced the results from the discovery-set panel and all 500 bootstrap runs. Repeated nested validation yielded an average AUC of 0.611 across 18 complete repetitions (range 0.417–0.750); 78 out of 80 outer fits were successful (Fig 3F; S5 Table). The 0.925 estimate subtracts mean bootstrap optimism from the apparent AUC, whereas 0.611 evaluates test-fold patients held out from panel selection.

**Fig 3:**
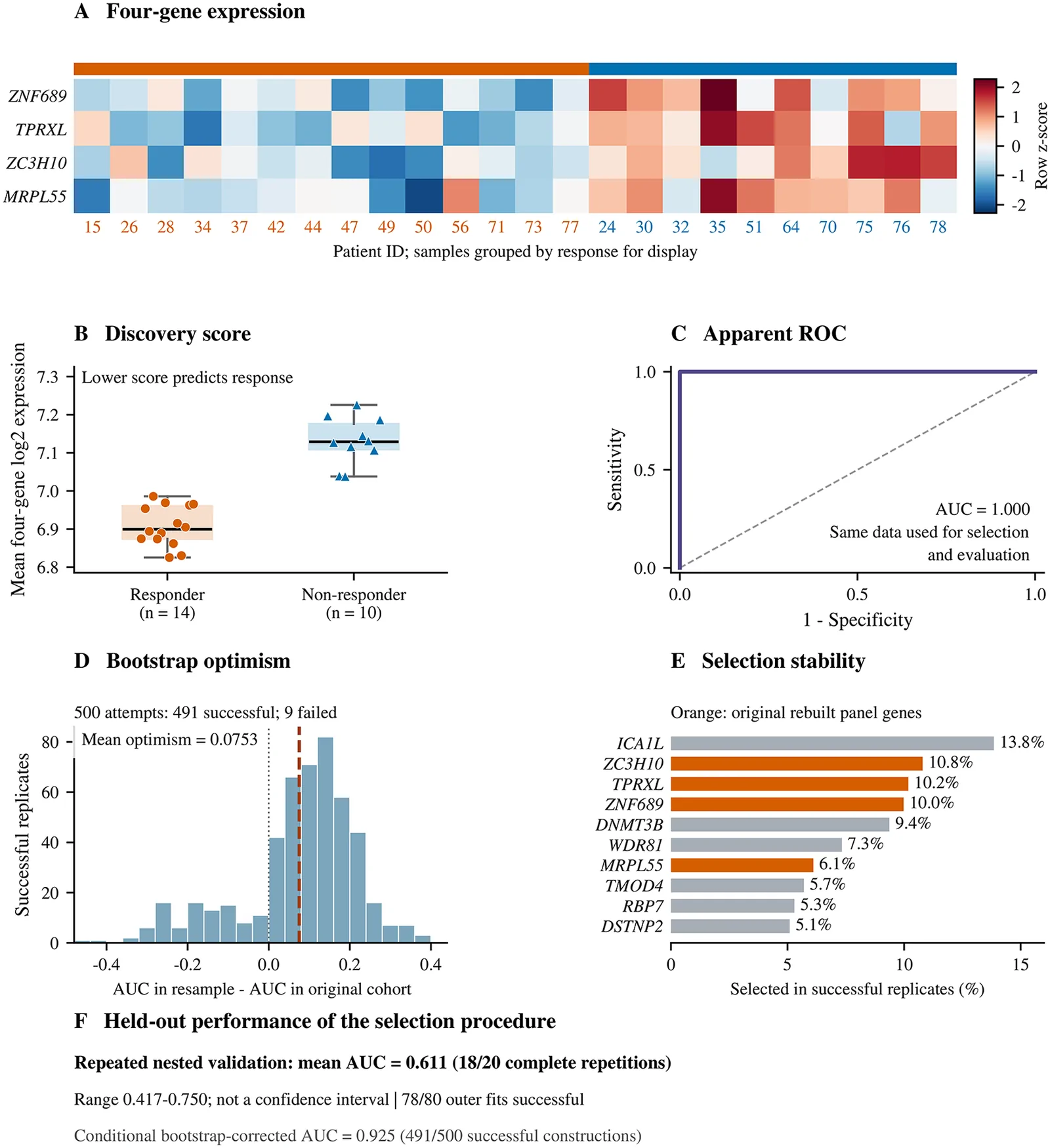
Discovery-stage performance and resampling instability. (A) Row-standardized expression of *ZNF689*, *TPRXL*, *ZC3H10* and *MRPL55*. (B) Mean log2 four-gene scores in 14 responders and ten non-responders. (C) Apparent ROC curve (AUC 1.000); its degenerate DeLong interval does not account for feature selection. (D) Optimism across 491 successful bootstrap panels from 500 attempts; nine attempts failed. Conditional corrected AUC = 0.925, without a confidence interval for that estimator. (E) Resampling selection frequencies. (F) Repeated nested validation: mean held-out AUC 0.611 across 18 complete repetitions, with 78/80 successful outer fits. The range 0.417–0.750 describes variation across splits, not a confidence interval. All panels derive from the same 24-patient discovery cohort. Samples are from GSE73661. Bootstrap and nested estimates use different training and evaluation conditions, with a fixed direction; these are internal exploratory estimates.

### Exploratory inflammatory pathway context

We evaluated three selected inflammatory panels by targeted GSVA; interferon-gamma provided the largest directional score difference. A negative effect estimate emerged in the responder-versus-non-responder contrast (mean GSVA-score difference −0.425, nominal *P* = 0.066; Benjamini–Hochberg-adjusted *P* = 0.197). This is a nonsignificant trend toward lower scores in responders, not evidence of reduced interferon-gamma function. Separation was weaker for both TNFα signaling and the inflammatory-response panel; neither reached significance (Fig 4). The panels each contain four specified genes and are not the complete Hallmark gene sets.

**Fig 4:**
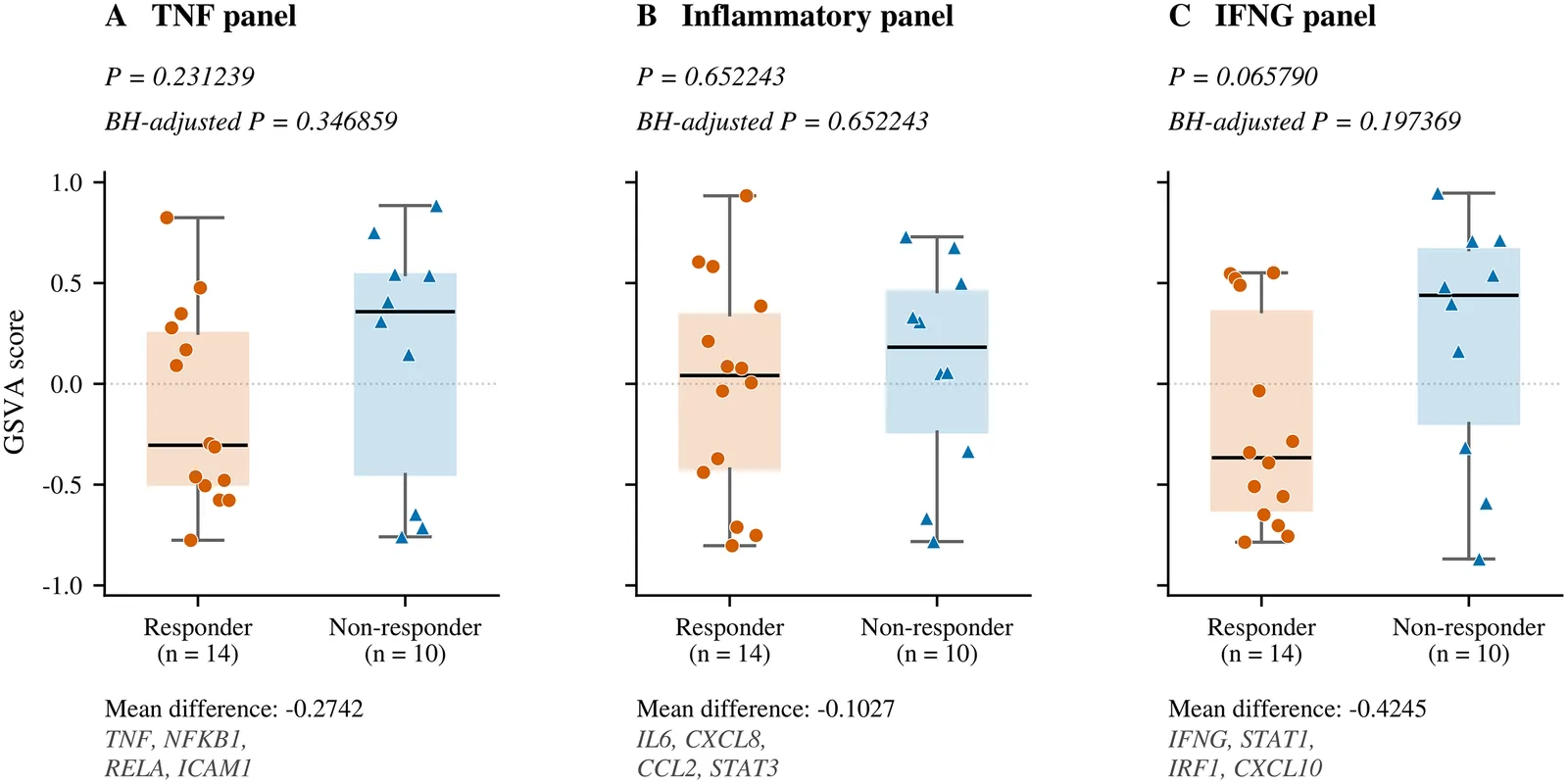
Exploratory targeted inflammatory-panel scores. GSVA scores in 14 responders and ten non-responders. Panels contain the four genes specified in Methods, not complete Hallmark gene sets. Two-sided limma nominal *P* values and Benjamini–Hochberg-adjusted *P* values across three panels are, respectively: TNF, 0.231/0.347; inflammatory response, 0.652/0.652; interferon-gamma, 0.066/0.197. No contrast achieved statistical significance. Boxes indicate quartiles and medians; whiskers extend to observations within 1.5 interquartile ranges. Plotted points represent individual samples. Samples are from GSE73661. Mean differences are responder minus non-responder.

### Exploratory projections in external blood and mucosal cohorts

We tested tissue-to-blood portability externally using an independent vedolizumab cohort of 17 baseline peripheral blood samples (9 in remission at week 14 and 8 not in remission). The AUC derived from the reconstructed four-gene panel was 0.458 (95% CI 0.162–0.755; *P* = 0.810; Fig 5A–B). In the subgroup consisting solely of patients with ulcerative colitis (UC), the AUC was 0.500 (6 in remission, 4 not in remission; 95% CI 0.066–0.934; *P* = 1.000). TPRXL had zero measured TPM in 14 of the 17 blood samples, which limited the comparability of the scores.

The same fixed gene set was projected onto 27 baseline UC mucosal samples from E-MTAB-7845 (16 responders and 11 non-responders, as defined by week-14 endoscopic remission in the source publication). The resulting AUC was 0.534 (95% CI 0.293–0.775; *P* = 0.786; Fig 5C–5D). The AUCs for the inception and validation subcohorts were 0.648 and 0.333, respectively, and both had wide confidence intervals (Fig 5E). The source publication describes these RNA-seq cohorts separately from the historical GSE73661 cohort [3]. These analyses did not demonstrate discriminatory power in the mucosal samples. The mean of the raw TPM values was inconsistent with the log2 expression scores used in the discovery set; therefore, these results cannot be used to validate a calibrated model. The AUCs for the previously submitted combination including TVP23C were 0.636 (95% CI 0.422–0.850; *P* = 0.246) in mucosa and 0.514 in blood. If the average log2(TPM + 1) is used for calculation instead, the AUCs for the original submission and the reconstructed combination in the mucosal data were 0.608 and 0.517, respectively. These sensitivity analyses did not demonstrate discrimination on external data (S5 Table).

**Fig 5:**
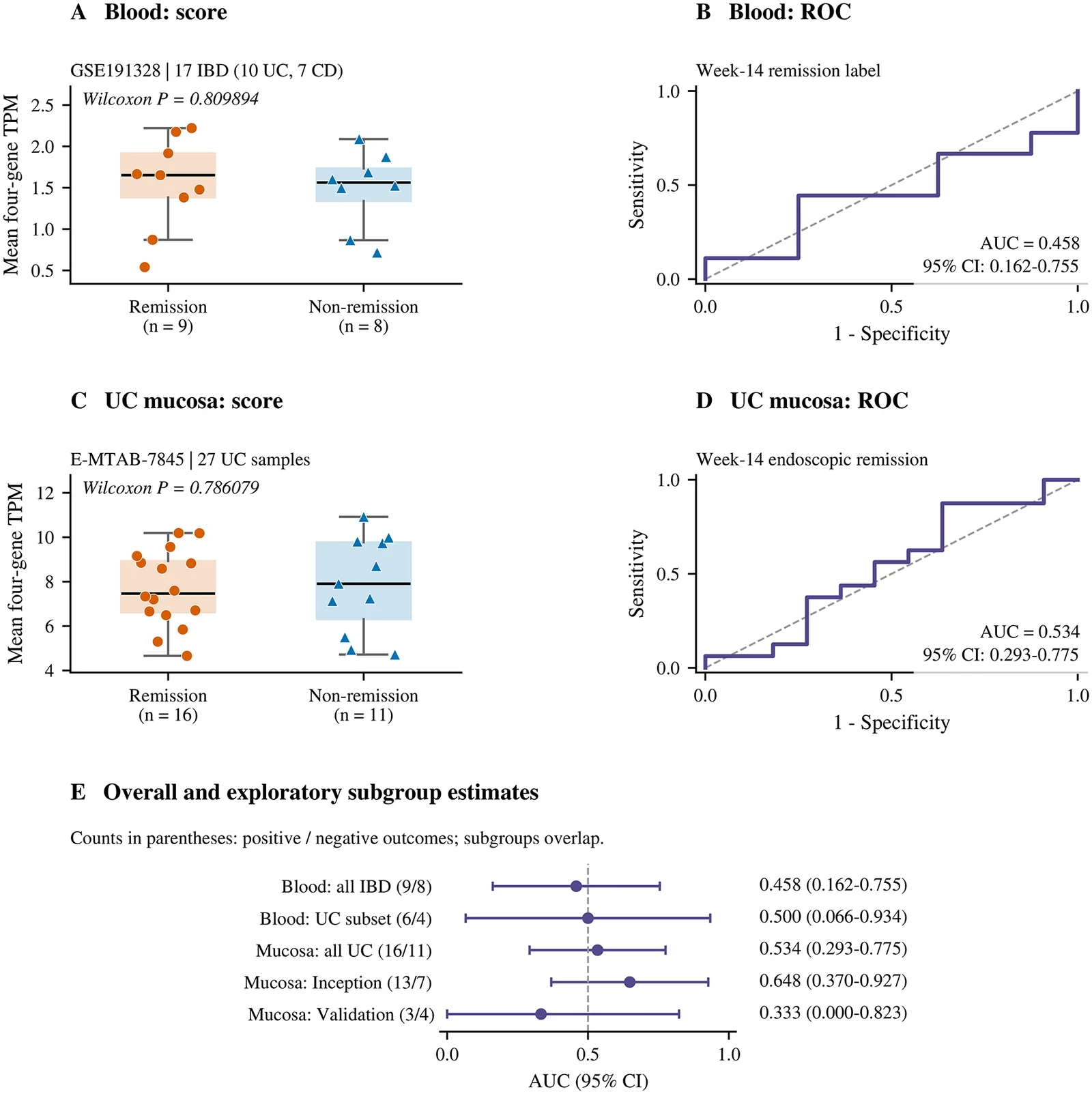
Exploratory application of the fixed gene set across different cohorts. (A–B) Scores and ROC curves in the GSE191328 blood cohort (including 9 patients in remission at week 14 and 8 patients not in remission). (C–D) Scores and ROC curves in the E-MTAB-7845 ulcerative colitis mucosal cohort (including 16 patients with week-14 endoscopic remission and 11 patients without remission). (E) AUC and its approximate 95% DeLong confidence interval: 0.458 (0.162–0.755) for the overall IBD blood cohort; 0.500 (0.066–0.934) for the UC-only blood subgroup (6/4); 0.534 (0.293–0.775) for overall mucosal UC; 0.648 (0.370–0.927) for the Inception subcohort (13/7); and 0.333 (0–0.823) for the Validation subcohort (3/4). The corresponding *P* values from the two-sided asymptotic Wilcoxon test were 0.810, 1.000, 0.786, 0.303, and 0.596, respectively. In A and C, the box in the boxplots represents the median and quartiles, with whiskers extending to values within 1.5 times the interquartile range; each point represents a single patient. The subgroups overlapped with the overall cohort, and the data were not combined for analysis. Scores were calculated as the average of the raw gene TPM values, with lower values indicating a predicted treatment response or remission; this differs from the log2 expression scale used in the discovery set. Throughout the analysis, parameters were not refitted on external data, nor was the prediction direction reversed. The fixed gene set comprises *ZNF689*, *TPRXL*, *ZC3H10* and *MRPL55*. Outcome definitions also differ from discovery. Mucosal Inception and Validation labels denote source-study subgroups.

### Biological context and study boundaries

See S3 Fig for descriptive correlation analyses that examined the associations between the signature genes and both treatment response and interferon-γ, respectively. In this reconstruction analysis, all four genes were negatively correlated with the composite response. The Spearman correlation coefficient between the mean values of these four genes and the interferon-γ score was 0.205 (S4 Fig). Being post-selection relationships, these correlations provide neither independent validation nor mechanistic insight.

Time-point sensitivity analysis was conducted using a reconstructed fixed gene set on the overlapping subcohort from the discovery set. At week 12 (13 patients total, including 3 responders and 10 non-responders), the AUC was 0.867 (95% CI 0.657–1.000; *P* = 0.076); at week 52 (19 patients total, including 13 responders and 6 non-responders), the AUC was 1.000. After excluding the vdz_plac subgroup from the source data, 17 patients remained (including 12 responders and 5 non-responders), and the AUC was also 1.000. The above analyses are all in-sample sensitivity assessments conducted after variable selection and do not constitute separate validation cohorts. Limiting the baseline Mayo endoscopic subscore to 2–3 points does not eliminate confounding effects resulting from residual disease severity or differences in cellular composition. Table 2 summarizes these results.

**Table 2:** Discovery discrimination, pathway context and contextual checks.

Metric	Value
Reconstructed discovery cohort	24 UC; 14 responders/10 non-responders; 18,865 genes
Rebuilt four-gene panel	*ZNF689*, *TPRXL*, *ZC3H10*, *MRPL55*; apparent AUC 1.000
Internal fixed-panel evaluation	Same-sample, post-selection estimate; degenerate DeLong CI 1.000–1.000. Previous fixed-panel bootstrap/permutation results withdrawn.
Full-pipeline bootstrap	500 attempts; 491 successful; nine failed. Mean optimism 0.075; conditional optimism-corrected AUC 0.925. Internal estimate, not externally validated; no CI for corrected estimator. Independent re-fitting reproduced all construction outcomes.
Repeated nested validation	20 repetitions of four-fold outer validation; 78/80 outer fits successful; 18 complete repetitions. Mean pair-weighted held-out AUC 0.611; median 0.625; range 0.417–0.750 (not a CI). Internal validation of the selection procedure; conditional on complete repetitions; S5 Table.
Blood all IBD, n = 17 (9/8)	AUC 0.458 (95% CI 0.162–0.755); *P* = 0.810
Blood UC only, n = 10 (6/4)	AUC 0.500 (95% CI 0.066–0.934); *P* = 1.000
Independent mucosal UC, n = 27 (16/11)	AUC 0.534 (95% CI 0.293–0.775); *P* = 0.786
Mucosal source subcohorts	Inception, 20 (13/7): AUC 0.648 (0.370–0.927), *P* = 0.303. Validation, 7 (3/4): AUC 0.333 (0–0.823), *P* = 0.596.
Interferon-gamma GSVA contrast	Mean score difference −0.425; nominal *P* = 0.066; BH-adjusted *P* = 0.197. Not statistically significant.
Discovery endpoint sensitivities	W12, 13 (3/10): AUC 0.867 (0.657–1), *P* = 0.076. W52, 19 (13/6): AUC 1 (1–1), *P* < 0.001. W52 excluding vdz_plac, 17 (12/5): AUC 1 (1–1), *P* = 0.002. Overlapping, in-sample analyses.

Note. All AUCs retain the fixed lower-score-predicts-response direction. External intervals are approximate 95% DeLong intervals, bounded to 0–1. *P* values are two-sided Wilcoxon rank-sum tests with asymptotic, continuity-corrected inference, except the limma pathway tests. The corrected AUC is internal and conditional on 491 successful bootstrap constructions; no confidence interval for this corrected estimator is available. Nested-validation repetitions reuse patients; their range is not a confidence interval. Bootstrap and nested estimates use different training and evaluation conditions.

## Discussion

In our reanalysis of public datasets, the selected four-gene combination was able to distinguish the response group to vedolizumab in the discovery cohort but failed to reliably distinguish the response group in external blood or mucosal samples. Although the apparent discrimination of the fixed gene combination was complete, full-process resampling revealed variability in gene selection. The weak blood projection does not establish tissue restriction, and the independent mucosal projection did not demonstrate reliable discrimination.

Vedolizumab selectively blocks the directed homing of lymphocytes to intestinal tissue; therefore, it is biologically reasonable to use intestinal mucosal tissue directly as the baseline specimen prior to treatment, whereas peripheral whole blood samples are anatomically distant from the actual site of disease [1,6]. Specifically, Arijs et al. linked the drug’s therapeutic efficacy to histological healing and changes in mucosal gene expression [2]; Verstockt and Gazouli also demonstrated that baseline markers in the colon anticipate patients’ subsequent treatment benefits [3,4]. Although sampling at the mucosal level has a solid clinical rationale, our exploratory analysis in the E-MTAB-7845 cohort did not demonstrate discriminatory power for the reconstructed panel.

Among the three selected panels tested, interferon-gamma showed the strongest directional difference, with non-responders exhibiting higher baseline scores. However, neither its nominal nor adjusted *P* value established statistical significance. The small targeted panel cannot measure cytokine function or resolve cellular sources.

These four genes should be regarded only as candidate markers of mucosal status; their definitive molecular mechanisms have not yet been established. Among them, *MRPL55* encodes a mitochondrial ribosomal protein [16]; since the reconstructed panel no longer includes *TVP23C*, the previous indirect argument involving *TVP23B* and intestinal barrier function does not support this revised panel. Although *ZC3H10* is associated with mitochondrial and metabolic transcriptional regulation [17,18], this does not establish that it is involved in gut trafficking. Evidence linking *TPRXL* (and related homeobox genes) [19,20] or *ZNF689* [21,22] to intestinal inflammation is limited.

Mechanistic investigations of vedolizumab have recently provided depth to this interpretation. Linked to response are TH17-monocyte communication and adhesion through α4β7. Further features of response include gut-associated lymphoid tissue remodeling, CD4 + infiltrates, and stromal/mononuclear phagocyte states [8–14]. Although these effects partially overlap with those of anti-TNF therapy, a key point emerges: local tissue programs and the biology of cell migration likely contribute to vedolizumab response.

Precision medicine for inflammatory bowel disease (IBD) has moved beyond simple transcriptomic predictions to encompass the areas of treatment goal setting and dose optimization [7,23–25]. Deep-healing strategies emphasize outcomes that go beyond mere symptom relief [23], while the VERDICT protocol describes a prospective comparison of different treatment goals [24]. These publications provide context for treatment targets assessed using the Mayo endoscopic subscore; however, they do not demonstrate the validity of combining data from week 12 and week 52 for analysis. The MOVE-IT protocol describes a trial of model-informed dosing of vedolizumab and ustekinumab [25].

Our data showed that, when applied to blood, the fixed four-gene score performed close to random levels. However, changes occurred simultaneously in tissue, platform, case mix, and response definition. A restricted UC-only sensitivity analysis yielded no gains in discriminatory power, and because these factors varied together, it cannot isolate a standalone tissue effect. The low detection rate of TPRXL in blood and the uneven contribution of genes on the TPM scale represent additional technical limitations. These data do not suggest that blood biomarkers in general contain less information than mucosal biomarkers [6].

One of the main limitations of this study is that the discovery cohort was small; no transcripts passed FDR multiple-test correction. The four-gene score was both derived and evaluated on the exact same dataset. Therefore, the conditionally corrected AUC of 0.925 should not be interpreted as predictive capability on external data: this value excludes nine failed constructions from the resampling process, and gene selection was unstable. Recovering the original main and sensitivity analyses brought clarity to sample attribution, but we could not fully reproduce the model estimates from our prior submission. Changes to the annotation and screening processes also prevented us from attributing the numerical discrepancies solely to patient 64. A pragmatic response definition combined the observed week-12 or week-52 outcomes of Mayo endoscopic subscore 0–1. Sensitivity analyses expose the different time points but did not eliminate bias from missing outcomes or potential treatment changes. The complete separation observed in the week-52 subgroup was especially susceptible to selection effects, as the outcomes at that time point constituted part of the composite label used to select genes. Therefore, this should not be interpreted as indicating that maintenance-phase endpoints are superior to induction-phase endpoints.

Future research should involve a pre-specified study based on same-tissue validation in a larger cohort. Harmonized measurement scales and definitions of efficacy outcomes must be established before analyzing patient treatment responses. At the same time, it is essential to independently validate these candidate genes using tissue qPCR, immunostaining, or histological analysis. Comparing these transcriptomic data with functional mechanistic readouts, such as cell adhesion assays, would help us assess the biological relevance of this panel and determine whether it can provide added value compared to existing tissue biomarkers [9,13,14]. Our current findings are not sufficient to guide clinical drug selection or adjust the dosage of vedolizumab.

## Methods

### Study design and analytic overview

This retrospective transcriptomic biomarker study utilized existing public datasets. The study had two objectives: first, to identify baseline mucosal features associated with the efficacy of vedolizumab in patients with ulcerative colitis; and second, to determine whether these features could be transferred across cohorts or tissues. Our core evaluation included reconstruction and derivation from the discovery set, internal evaluation, and exploratory application of the fixed gene panel to peripheral blood and independent mucosal RNA-seq datasets. Supplementary analyses addressed pathway context and sensitivity at different follow-up time points. Mapping the study design and data analysis pipeline, Fig 1 details our overall approach.

### Public datasets and cohort construction

Although the public longitudinal IBD transcriptomic dataset GSE191328 was originally published to evaluate patients’ therapeutic responses to anti-TNF drugs, it included baseline vedolizumab samples that could be used for our independent challenge [26]. Filtering on week-14 clinical remission status yielded a baseline subset of 17 peripheral blood samples (10 ulcerative colitis, 7 Crohn’s disease). These outcome labels did not fully align with the composite mucosal outcome based on the Mayo endoscopic subscore used in this study; therefore, we used this dataset only as an exploratory test of portability, rather than as a validation cohort with fully consistent labeling.

E-MTAB-7845 supplied 27 baseline UC mucosal RNA-seq samples: 20 Inception samples (13 responders/seven non-responders) and seven Validation samples (three/four). The source UC endpoint was endoscopic remission, defined as Mayo endoscopic subscore ≤1 at week 14 after vedolizumab [3]. Twenty Crohn’s disease samples in the study metadata were excluded. Study-level cohort independence follows the source publication; identifying cross-study patient records were unavailable for direct deduplication. The analysis cohorts are summarised in Table 1.

### Expression processing and harmonisation

For the mucosal microarray cohort [2], we obtained preprocessed series-matrix expression data using the R package GEOquery [27]. We analyzed only baseline biopsy tissues from patients with ulcerative colitis who were treated with vedolizumab. During probe annotation, we used the R package hugene10sttranscriptcluster.db to convert probe IDs to gene symbols, retained only single-gene probe mappings, and averaged the expression levels of multiple probes corresponding to the same gene. The reconstructed discovery set expression matrix comprised 24 samples and 18,865 genes. The composite response criterion required patients to have a Mayo endoscopic subscore of 0–1 at either observed week-12 or week-52 assessment; missing measurements were not imputed. Principal component analysis (PCA) utilized centered but unscaled expression data, while hierarchical clustering employed the Euclidean distance matrix and the complete linkage algorithm. S2 Table provides the cohort inventory, tracking patient identifiers, observed endpoints, and sample retention.

We used the GEO-provided normalised TPM matrix for the external blood cohort. The four target symbols each mapped to one Entrez Gene identifier in the saved annotation. Finally, the dataset was filtered to include only baseline vedolizumab blood samples for the exploratory challenge.

### Gene-level differential expression and feature selection

Differential expression at the gene level comparing non-responders and responders was assessed using limma [28]. Due to our small cohort size and the lack of transcripts surviving multiple-testing adjustments, individual gene data served mainly to rank effects and guide exploratory prioritization.

Repeated LASSO feature selection was performed using glmnet [29]. The reconstructed screening procedure followed the workflow described in the submitted Methods, in contrast to the 24 leave-one-out selections and lambda.min parameter of the recovered historical code (S1 Methods). These are distinct workflows and do not constitute evidence of original pre-specification. Agreement with the description in the submitted manuscript neither resolves the discrepancies with the historical execution records nor establishes the superiority of the revised workflow. For the derivation analyses, the candidate genes ranked by limma were narrowed down to the top 200. LASSO selection was repeated 50 times using stratified five-fold cross-validation, binomial deviance, standardized predictors and lambda.1se. The final four genes were chosen by selection frequency across 50 repetitions; ties were resolved by limma rank. The discovery score was the unweighted mean log2 expression of *ZNF689*, *TPRXL*, *ZC3H10* and *MRPL55*, with lower values predicting response; it was not the fitted LASSO-weighted logistic score. No external sign-flipping, feature reselection or coefficient refitting was allowed. Preserving cross-validation folds alongside bootstrap draws permits independent checking of repeated-patient grouping and numerical evaluation.

### Exploratory pathway analysis

We used gene set variation analysis (GSVA) to evaluate mucosal signaling in the context of inflammatory pathways [30]. Rather than evaluating wide signaling networks, we narrowed our focus to three gene panels associated with mucosal inflammation, incorporating TNF-α signaling (TNF, NFKB1, RELA, ICAM1), the inflammatory response (IL6, CXCL8, CCL2, STAT3), and the interferon-γ response (IFNG, STAT1, IRF1, CXCL10). The limma package was then used to test for differences at the pathway level between responders and non-responders, with Benjamini–Hochberg correction applied across the three tests. The reported results are differences in GSVA scores, not log fold changes in gene expression.

### Internal evaluation and resampling

The fixed-panel evaluation measures the apparent discriminatory power (apparent AUC) on the dataset used to derive it. We conducted a full-pipeline bootstrap resampling: over 500 resampling cycles, we reconstructed the entire derivation process—including rerunning limma ranking, screening the top 200 genes, running LASSO 50 times, and selecting four genes. If a patient was sampled multiple times, all duplicate copies were kept within the same cross-validation fold; the number of folds was the minimum of five and the numbers of unique patients in the two outcome groups. While maintaining the fixed score direction, each reconstructed panel was tested separately on its own bootstrap sample and on the original 24 patients; the difference between these two AUCs was defined as optimism. The corrected AUC reported here was calculated by subtracting the mean optimism from the original apparent AUC, conditional on successful panel construction. We preserved failed construction attempts for later inspection. Inner-loop cross-validation was used to tune the LASSO hyperparameters. We additionally ran 20 rounds of repeated “4-fold stratified” outer-layer cross-validation. Recalculating feature priorities with each validation run, we performed gene ranking and all 50 LASSO screening rounds independently inside every new training subset. The held-out within-fold AUC was weighted by the total number of “responder/non-responder” patient pairs, and we did not combine scores calculated from different panels. Bootstrap and outer-layer nested testing are entirely different concepts: the former includes patients who have already been used to train the model when testing with the raw data, whereas the latter excludes test-fold patients from label-dependent ranking and panel selection. Earlier processed-array normalization and annotation were not re-estimated within folds. Nested validation evaluates the selection procedure, not the fixed final four-gene panel; its summary is conditional on the 18 complete repetitions.

### Exploratory cross-platform projections

External evaluation applied the rebuilt four-gene set without modification. Blood and E-MTAB-7845 scores were the arithmetic mean of four gene-level TPM values; all four genes were required. Discrimination and score separation were quantified using ROC curves, AUC estimates, and Wilcoxon rank-sum tests. Gene membership and direction were fixed, but TPM averaging is not measurement-equivalent to averaging microarray log2 expression. These analyses therefore examine exploratory cross-platform portability, without calibrated thresholds. Blood UC-only and source-defined mucosal subcohorts were reported separately without selecting the best-performing subset. Sensitivity analyses applied both the submitted and rebuilt panels using raw TPM and log2(TPM + 1), retaining all cohort results and the fixed direction.

### Interpretive analyses

Pairwise Spearman correlations described relationships among the four signature targets, the interferon-gamma GSVA score, and clinical response (responder = 1). A four-gene set this sparse and functionally mixed cannot support stable pathway-level inference, so we did not attempt formal over-representation analysis. Biological interpretation relied on documented literature annotations.

For E-MTAB-7845, SRA-formatted sequences were converted to FASTQ format and processed using fastp 0.23.4 [31] with the specified TruSeq adapters. Salmon 1.10.2 [32] was used to perform quantitative analysis on the single-end sequencing reads based on GENCODE v47 transcripts [33], using primary-genome decoys and a k value of 23. The raw read length was 51 bases, and Salmon inferred a reverse-strand single-end library for each sample. Quantitative analysis was completed for all 27 samples; the mapping rates ranged from 90.04% to 94.55%. Gene TPM values were calculated by summing the transcript TPM values. TPRXL maps to two Ensembl gene identifiers; the main analysis sums both, while sensitivity analyses based on a single identifier yielded UC AUC values identical to those of the main analysis. The mean and standard deviation of single-end fragment lengths were assumed to be 200 and 80 bases, respectively; these were not actually measured. The S1 Methods section tabulates command sequences, annotation protocols, and study limitations.

In the sensitivity analysis of endpoints, the reconstructed discovery set panel remained fixed, and patients with missing outcomes at each follow-up time point were excluded only from the corresponding specific analysis. After excluding the treatment category vdz_plac (vedolizumab/placebo group) from the original dataset, we repeated the Week 52 analysis.

### Ethics statement and AI use

This study re-analysed de-identified public datasets and did not involve new participant recruitment, intervention or specimen collection. Under local regulations, additional institutional review board approval and informed consent were therefore not required. DeepL assisted during manuscript revision with language editing. Computational outputs were compared with recovered source records and separately executed R analyses; these checks do not constitute independent human verification. The tool did not generate original observations or independently determine scientific conclusions. The authors remain responsible for verifying the data, analyses, figures and interpretations and for reviewing and approving the final submission.

### Statistical analysis

Source statistical analyses used R version 4.5.2 [34], limma [28], GSVA [30], glmnet [29] and pROC [35]; revision-stage numerical checks and figure preparation also used Python. External AUC intervals use the DeLong variance with a normal approximation bounded to 0–1. Wilcoxon tests are two-sided, asymptotic and continuity-corrected. Nominal *P* values are reported numerically; exploratory external subgroup comparisons were not multiplicity-adjusted. Complete in-sample separation produces degenerate intervals and is not evidence of certainty. Independent re-execution used R 4.3.3, limma 3.58.1 and glmnet 4.1–8. Probe aggregation, discovery selection and 500 bootstrap constructions were checked against source objects.

## Supporting information

S2 Table Sample-level audit of the reconstructed discovery cohort. The table documents all 24 GSE73661 baseline samples (14 composite responders and 10 non-responders), observed week-12/week-52 Mayo endpoints, treatment codes, retention and mean log2 four-gene scores. Missing outcomes were not imputed. Patient 64 was retained. Historical inclusion records identify a 24-sample main analysis and a separate sensitivity excluding patient 64. The exclusion operation is recovered; its preset technical justification and connection to all submitted 23-sample estimates remain unverified.
(DOCX)

S1 Methods Supplementary methods and analysis provenance. Cohort reconstruction, resampling, RNA-seq processing, external endpoints, independent re-execution and remaining historical limitations.
(DOCX)

S1 Data Analysis code, numerical tables and RNA-seq data. Sample-level scores, RNA-seq gene TPM and processing metadata, figure source tables, re-fitting outputs and executable analysis scripts. The root README, DATA_ARCHIVES.csv and SHA256SUMS.txt describe the five archives and their common directory structure.
(ZIP)

S2 Data Discovery expression and processed-probe checkpoints. The complete processed-probe audit and reconstructed discovery expression, annotation and selection objects under checkpoints.
(ZIP)

S3 Data Resampling and pathway checkpoints. Bootstrap, pathway and companion checkpoints, together with the reconstruction input used by the independent R analysis. Original relative paths are preserved.
(ZIP)

S4 Data Independent R source inputs. The processed-probe audit, full bootstrap input and summary input under Revision2/inputs. Extract S1–S5 Data into the same directory before running the supplied scripts.
(ZIP)

S3 Fig Descriptive correlations within the reconstructed discovery cohort. Spearman correlations among four-gene log2 expression, the targeted *IFNG*-panel score and composite response (1 = responder, 0 = non-responder) in 24 samples (14/10). Cell labels show correlation coefficients. These post-selection associations are descriptive, with no significance tests or independent-validation claim.
(DOCX)

S4 Fig Relationship between the rebuilt four-gene score and the targeted *IFNG*-panel score. Each point represents one of 24 discovery samples (14 responders/10 non-responders). Spearman rho is 0.205. This post-selection companion display does not establish incremental prediction, a combined model or external validity, and is not a co-primary model.
(DOCX)

S5 Table Analysis reconstruction and revision-stage sensitivity checks. Historical and reconstructed panel/annotation comparison, fixed-direction external panel/scale results, independent R re-fitting and repeated nested validation. These added sensitivities were not prospectively specified and do not replace the primary analysis.
(DOCX)

S5 Data Recovered historical analysis provenance. Sample-level main-versus-sensitivity inclusion, patient 64 follow-up, original probe mappings, historical selection frequencies, source code and numerical checks. These records distinguish the recovered 24-sample main analysis and 23-sample exclusion sensitivity from later submitted model estimates that remain unmatched. Verification against original R objects requires the source files identified by checksum; current-panel evidence is linked separately.
(ZIP)
