# Supplementary material for: Exploratory baseline mucosal transcriptomics and vedolizumab response in ulcerative colitis
Source: PLoS One. 2026 Oct 26;21(10):e0361301. doi: 10.1371/journal.pone.0361301 (PMC13705592; doi:10.1371/journal.pone.0361301)
Supplement: Supplementary file 1 [file pone.0361301.s001.docx]

S2 Table. Sample-level audit of the reconstructed discovery cohort

The table documents all 24 GSE73661 baseline samples (14 composite responders and 10 non-responders), observed week-12/week-52 Mayo endpoints, treatment codes, retention and mean log2 four-gene scores. Missing outcomes were not imputed. Patient 64 was retained. Historical inclusion records identify a 24-sample main analysis and a separate sensitivity excluding patient 64. The exclusion operation is recovered; its preset technical justification and connection to all submitted 23-sample estimates remain unverified.

# S2_samples — clinical outcomes and score

| **Patient** | **GSM** | **Treatment** | **Mayo_W12** | **Mayo_W52** | **Response_audit** | **Rebuilt_four_gene_score** |
| --- | --- | --- | --- | --- | --- | --- |
| 15 | GSM1900227 | vdz_vdz8w |  | 0 | Responder | 6.90500860225 |
| 24 | GSM1900317 | vdz4w | 3 | 3 | Non-responder | 7.131071589 |
| 26 | GSM1900325 | vdz4w | 3 | 1 | Responder | 6.96529696775 |
| 28 | GSM1900236 | vdz_vdz4w |  | 0 | Responder | 6.889316957 |
| 30 | GSM1900263 | vdz_plac |  | 2 | Non-responder | 7.144590374 |
| 32 | GSM1900318 | vdz4w | 3 |  | Non-responder | 7.03916429475 |
| 34 | GSM1900265 | vdz_vdz4w |  | 1 | Responder | 6.874161702 |
| 35 | GSM1900323 | vdz4w | 2 | 2 | Non-responder | 7.2256419345 |
| 37 | GSM1900313 | vdz4w | 0 | 0 | Responder | 6.962404099 |
| 42 | GSM1900271 | vdz_vdz4w |  | 1 | Responder | 6.9152966415 |
| 44 | GSM1900314 | vdz4w | 1 |  | Responder | 6.95357744025 |
| 47 | GSM1900277 | vdz_vdz4w |  | 0 | Responder | 6.89413649425 |
| 49 | GSM1900280 | vdz_vdz4w |  | 0 | Responder | 6.83044833 |
| 50 | GSM1900268 | vdz_vdz4w |  | 0 | Responder | 6.8254242755 |
| 51 | GSM1900319 | vdz4w | 2 |  | Non-responder | 7.116017863 |
| 56 | GSM1900321 | vdz4w | 0 | 1 | Responder | 6.9858893665 |
| 64 | GSM1900320 | vdz4w | 3 | 3 | Non-responder | 7.18618500175 |
| 70 | GSM1900324 | vdz4w | 3 |  | Non-responder | 7.038097528 |
| 71 | GSM1900322 | vdz4w | 3 | 0 | Responder | 6.87483648375 |
| 73 | GSM1900260 | vdz_plac |  | 0 | Responder | 6.8620606515 |
| 75 | GSM1900315 | vdz4w | 3 |  | Non-responder | 7.1966795835 |
| 76 | GSM1900294 | vdz_vdz4w |  | 2 | Non-responder | 7.126912583 |
| 77 | GSM1900302 | vdz_vdz4w |  | 1 | Responder | 6.96896137875 |
| 78 | GSM1900316 | vdz4w | 2 | 3 | Non-responder | 7.1069095785 |

# S2_samples — inclusion and quality audit

| **Patient** | **Included_rebuilt_cohort** | **Included_W12** | **Included_W52** | **Included_W52_without_vdz_plac** | **Nonfinite_gene_expression_values** | **PCA_review_note** |
| --- | --- | --- | --- | --- | --- | --- |
| 15 | TRUE | FALSE | TRUE | TRUE | 0 |  |
| 24 | TRUE | TRUE | TRUE | TRUE | 0 |  |
| 26 | TRUE | TRUE | TRUE | TRUE | 0 |  |
| 28 | TRUE | FALSE | TRUE | TRUE | 0 |  |
| 30 | TRUE | FALSE | TRUE | FALSE | 0 |  |
| 32 | TRUE | TRUE | FALSE | FALSE | 0 |  |
| 34 | TRUE | FALSE | TRUE | TRUE | 0 |  |
| 35 | TRUE | TRUE | TRUE | TRUE | 0 |  |
| 37 | TRUE | TRUE | TRUE | TRUE | 0 |  |
| 42 | TRUE | FALSE | TRUE | TRUE | 0 |  |
| 44 | TRUE | TRUE | FALSE | FALSE | 0 |  |
| 47 | TRUE | FALSE | TRUE | TRUE | 0 |  |
| 49 | TRUE | FALSE | TRUE | TRUE | 0 |  |
| 50 | TRUE | FALSE | TRUE | TRUE | 0 |  |
| 51 | TRUE | TRUE | FALSE | FALSE | 0 |  |
| 56 | TRUE | TRUE | TRUE | TRUE | 0 |  |
| 64 | TRUE | TRUE | TRUE | TRUE | 0 | Visually separated in PCA; retained |
| 70 | TRUE | TRUE | FALSE | FALSE | 0 |  |
| 71 | TRUE | TRUE | TRUE | TRUE | 0 |  |
| 73 | TRUE | FALSE | TRUE | FALSE | 0 |  |
| 75 | TRUE | TRUE | FALSE | FALSE | 0 |  |
| 76 | TRUE | FALSE | TRUE | TRUE | 0 |  |
| 77 | TRUE | FALSE | TRUE | TRUE | 0 |  |
| 78 | TRUE | TRUE | TRUE | TRUE | 0 |  |

# Definitions — analysis sets and fields

| **Analysis set** | **N** | **Responders** | **Non-responders** |
| --- | --- | --- | --- |
| Composite | 24 | 14 | 10 |
| Week 12 | 13 | 3 | 10 |
| Week 52 | 19 | 13 | 6 |
| Week 52 excluding vdz_plac | 17 | 12 | 5 |

## Interpretation and missing data

Composite response: Mayo endoscopic subscore 0 or 1 at either observed week-12 or week-52 assessment. Timepoint-specific counts use that timepoint only.

Blank Mayo cells are missing observations, not zero. No missing outcome was imputed. The analysis sets overlap and must not be added together.

Recovered records identify a 24-sample post-merge main analysis (14 responders/10 non-responders) and a 23-sample sensitivity analysis excluding patient 64 (14/9). The deletion is documented; its objective criterion and the link to later submitted 23-sample model estimates remain unresolved. See Historical_inclusion and S5 Data.

Patient 64 was retained. Visual separation in PCA alone was not used as an exclusion criterion.

Zero nonfinite expression values describes the processed 18,865-gene matrix. This is not a raw-array quality-control result.

The rebuilt panel is ZNF689, TPRXL, ZC3H10 and MRPL55. It differs from the historical panel; the score is not an independent validation result.

## Field definitions (original source columns retained)

| **Field** | **Definition** |
| --- | --- |
| Patient | Patient identifier in the source sample metadata. |
| GSM | GEO sample accession; one baseline sample per patient. |
| Treatment | Treatment code preserved verbatim from the source metadata. |
| Mayo_W12 | Observed week-12 Mayo endoscopic subscore; blank = missing. |
| Mayo_W52 | Observed week-52 Mayo endoscopic subscore; blank = missing. |
| Response_audit | Composite responder/non-responder classification defined above. |
| Included_rebuilt_cohort | TRUE means included in the reconstructed 24-sample cohort. |
| Included_W12 | TRUE means week-12 Mayo outcome is observed. |
| Included_W52 | TRUE means week-52 Mayo outcome is observed. |
| Included_W52_without_vdz_plac | TRUE means week-52 outcome observed and Treatment is not vdz_plac. |
| Nonfinite_gene_expression_values | Count of nonfinite values in the processed 18,865-gene expression matrix. |
| Rebuilt_four_gene_score | Arithmetic mean log2 expression of ZNF689, TPRXL, ZC3H10 and MRPL55; full numeric precision retained. |
| PCA_review_note | Source PCA review note; blank means no note recorded, not proof of absence of other quality issues. |

## Provenance and scope

Source: S2_sample_audit_24.csv; cross-checked with VDZ_S2_sample_audit_24.rds.

Expression and metadata cross-check: VDZ_rebuild_before_bootstrap_20260915_055133.rds. See Supplement_audit.json for SHA-256 hashes.

This table covers the reconstructed discovery cohort only, not E-MTAB-7845 or a complete screening log of all source-study samples.

# Historical_inclusion — 24-patient main set and patient 64 sensitivity

| **Patient** | **GSM** | **Response** | **Historical main (24)** | **Sensitivity excl. 64 (23)** | **Historical exclusion detail** |
| --- | --- | --- | --- | --- | --- |
| 15 | GSM1900227 | Responder | 1 | 1 | Included in both historical sets. |
| 24 | GSM1900317 | Non-responder | 1 | 1 | Included in both historical sets. |
| 26 | GSM1900325 | Responder | 1 | 1 | Included in both historical sets. |
| 28 | GSM1900236 | Responder | 1 | 1 | Included in both historical sets. |
| 30 | GSM1900263 | Non-responder | 1 | 1 | Included in both historical sets. |
| 32 | GSM1900318 | Non-responder | 1 | 1 | Included in both historical sets. |
| 34 | GSM1900265 | Responder | 1 | 1 | Included in both historical sets. |
| 35 | GSM1900323 | Non-responder | 1 | 1 | Included in both historical sets. |
| 37 | GSM1900313 | Responder | 1 | 1 | Included in both historical sets. |
| 42 | GSM1900271 | Responder | 1 | 1 | Included in both historical sets. |
| 44 | GSM1900314 | Responder | 1 | 1 | Included in both historical sets. |
| 47 | GSM1900277 | Responder | 1 | 1 | Included in both historical sets. |
| 49 | GSM1900280 | Responder | 1 | 1 | Included in both historical sets. |
| 50 | GSM1900268 | Responder | 1 | 1 | Included in both historical sets. |
| 51 | GSM1900319 | Non-responder | 1 | 1 | Included in both historical sets. |
| 56 | GSM1900321 | Responder | 1 | 1 | Included in both historical sets. |
| 64 | GSM1900320 | Non-responder | 1 | 0 | ID-specific deletion; objective criterion not recovered. |
| 70 | GSM1900324 | Non-responder | 1 | 1 | Included in both historical sets. |
| 71 | GSM1900322 | Responder | 1 | 1 | Included in both historical sets. |
| 73 | GSM1900260 | Responder | 1 | 1 | Included in both historical sets. |
| 75 | GSM1900315 | Non-responder | 1 | 1 | Included in both historical sets. |
| 76 | GSM1900294 | Non-responder | 1 | 1 | Included in both historical sets. |
| 77 | GSM1900302 | Responder | 1 | 1 | Included in both historical sets. |
| 78 | GSM1900316 | Non-responder | 1 | 1 | Included in both historical sets. |

## Historical analysis set

| **Analysis set** | **N** | **Responders** | **Non-responders** |
| --- | --- | --- | --- |
| Main after outcome merge | 24 | 14 | 10 |
| Sensitivity excluding 64 | 23 | 14 | 9 |

## Patient 64 endoscopic Mayo scores

| **Patient** | **GSM** | **Assessment** | **Mayo endoscopic subscore** |
| --- | --- | --- | --- |
| 64 | GSM1900320 | W0 | 3 |
| 64 | GSM1900171 | W12 | 3 |
| 64 | GSM1900200 | W52 | 3 |

Historical main: 24 samples after patient-level outcome merging and exclusion of missing responses. Sensitivity: the same matrix with patient 64 removed.

Patient 64 (GSM1900320) had Mayo scores of 3 at both weeks 12 and 52. Missing outcome does not explain the historical exclusion.

The ID-specific deletion is recovered. No prespecified objective technical exclusion criterion or complete link to the later submitted 23-sample model estimates was recovered.

Current inclusion, outcomes and four-gene scores remain as recorded in S2_samples. These historical fields document provenance and do not change the revised primary cohort.

Inclusion flags: 1 = included; 0 = excluded. Response labels follow the recovered historical metadata.

Source: S5_Data_V8_Historical_Provenance.zip, historical_exports/sample_24_vs_23.csv and historical_exports/patient64_followup.csv.

Original records: VDZ_workspace.RData (model_meta2, model_meta_sens) and the R console record, lines 1344–1354 and 1802–1815. See S5 Data, historical_exports/source_code_excerpts.txt.

Formula-derived cells above show the saved calculated values from the source workbook; Word does not execute the original Excel formulas.
