# Supplementary material for: Exploratory baseline mucosal transcriptomics and vedolizumab response in ulcerative colitis
Source: PLoS One. 2026 Oct 26;21(10):e0361301. doi: 10.1371/journal.pone.0361301 (PMC13705592; doi:10.1371/journal.pone.0361301)
Supplement: Supplementary file 2 [file pone.0361301.s002.docx]

S1 Methods Supplementary methods and analysis provenance

Cohort reconstruction, resampling, RNA-seq processing, external endpoints, independent re-execution and remaining historical limitations.

PONE-D-26-17559 | Companion to the revised manuscript and S1–S5 Data

# M1 Scope of the reconstructed analysis

This supplement describes the revised analysis and the numerical resources supplied in S1–S5 Data. The reconstruction is not a replication of every submitted 23-sample result. Recovered historical records distinguish a 24-sample main analysis from a 23-sample sensitivity analysis excluding patient 64 (GSM1900320). An objective prespecified technical exclusion criterion was not recovered. The revised primary analysis retains all 24 documented baseline UC samples. Single-gene probe annotation yielded 18,865 genes, and the reconstructed panel comprises *ZNF689*, *TPRXL*, *ZC3H10* and *MRPL55*. The earlier fixed-panel bootstrap/permutation estimates and top-10/top-20 blood transfers are not mixed with these results. The submitted *TVP23C*-containing panel is separately evaluated in external sensitivity analyses (S5 Table).

The recovered console record and saved objects identify the historical patient exclusion and matching 23-sample sensitivity matrix; they do not establish complete execution provenance for the later submitted 23-sample model estimates. During this revision, the complete 24-sample probe audit was restored from its original ZIP entry, and the bootstrap function embedded in the supplied full RDS was recovered using R. Independent code was then executed from the processed probe matrix and saved annotation through gene aggregation, discovery selection and all 500 bootstrap constructions. These steps extend beyond numerical replay of the reconstructed analysis; they do not reprocess raw microarray CEL files or rerun the complete historical model pipeline.

The submitted manuscript described the 23-sample set as a post-merge matrix. Recovered code instead shows 24 samples after baseline–outcome merging, followed by a separate exclusion of patient 64 for sensitivity analysis. The link between that sensitivity analysis and every later submitted 23-sample estimate remains unverified (M14).

# M2 Discovery samples and clinical endpoints

S2 Table provides GSM accession, patient identifier, treatment code, observed week-12 and week-52 endoscopic Mayo subscores, composite response, retention fields and the rebuilt four-gene score for each patient. Composite response is an observed Mayo endoscopic subscore of 0 or 1 at either timepoint. Missing outcome observations are not replaced by nonresponse or imputed. All 24 patients have the composite label used in the saved analysis: 14 responders and ten non-responders. Baseline endoscopic subscores range from 2 to 3; this does not remove confounding by severity, cell mixture or other clinical factors.

Patient 64 was retained despite visual separation in PCA. The historical sensitivity analysis excluded this patient, although both W12 and W52 endoscopic Mayo subscores were 3 and no prespecified technical exclusion threshold was recovered. The available processed matrix contains complete numerical expression values; this is not equivalent to a new raw-array quality-control assessment. The source vdz_plac treatment label is retained verbatim and is removed only in the specified week-52 sensitivity. The source study’s narrative description of historical follow-up must not be used to relabel the deposited W12 field as W14. The discovery W12/W52 fields and external W14 endpoint remain distinct.

# M3 Expression preparation and feature selection

GSE73661 processed series-matrix values were imported with GEOquery. Annotation used hugene10sttranscriptcluster.db. For the revision, only probes mapping to one gene symbol were retained, and multiple eligible probes for the same gene were averaged on the processed log2-expression scale. The resulting matrix has 18,865 rows and 24 columns. PCA centers each gene and does not divide by its standard deviation. Hierarchical clustering uses Euclidean sample distance and complete linkage. Neither display determines exclusion. The uniform rule avoids assigning a multiply mapped probe exclusively to one gene; it is a revision-stage annotation choice.

Limma ranked genes by the responder-versus-non-responder comparison; no single gene survived false-discovery adjustment. The 200 top-ranked candidates entered 50 repeated LASSO fits, with binomial deviance, alpha = 1, standardized predictors, stratified five-fold cross-validation and lambda.1se. Genes were ranked by the number of fits with nonzero coefficients; equal frequencies retained the limma order. *ZNF689*, *TPRXL*, *ZC3H10* and *MRPL55* were selected in 41, 38, 36 and 20 of the 50 fits. *MRPL55* and SIRPD were each selected in 20 fits; *MRPL55* was retained because it ranked earlier in the limma ordering (23rd versus 55th). The score is their arithmetic mean log2 expression, with lower scores predicting response. It is not the fitted weighted logistic linear predictor. The within-fit cross-validation tunes the penalty; it is not a separate, unbiased performance evaluation of the entire selection process. This implements the top-200, 50-repeat, lambda.1se workflow described in the submitted Methods. The available execution records establish the procedure used, but do not establish original prespecification or the authors’ contemporaneous reasons for departing from the recovered historical algorithm. Agreement with the submitted description does not resolve the discrepancy with recovered execution records or establish superiority of the revised procedure.

The saved probe audit contains 33,252 probe rows. Of these, 19,871 have one eligible gene symbol and aggregate to 18,865 genes. Independent aggregation reproduced every entry of the saved gene matrix exactly and reproduced all 24 outcome labels. *TVP23C* has two associated probes: 8005501 maps to *TVP23B*, *TVP23C* and *TVP23C*-CDRT4; 8012924 maps to *TVP23C* and *TVP23C*-CDRT4. Both are excluded by the single-gene rule, so *TVP23C* is absent from the reconstructed discovery matrix. The recovered historical TVP23C measurement was the arithmetic mean of these two probes. This establishes an annotation-related change in candidate availability; the MRPL55-containing panel was selected anew under the revised procedure, rather than produced by manually substituting one gene.

# M4 Full-pipeline bootstrap and internal uncertainty

Each of 500 bootstrap attempts samples 24 patient indices with replacement, repeats limma ranking, top-200 filtering and 50 LASSO fits, and constructs a four-gene panel by frequency. Duplicate copies of one patient stay in the same internal fold. The successful records contain 457 attempts with five folds, 32 with four and two with three, reflecting the available unique patients within outcome classes. Saved fold assignments permit direct verification.

For each successful attempt, the newly selected panel is scored by the same mean-expression rule in the bootstrap sample and the original 24 patients. Optimism equals bootstrap AUC minus original-cohort AUC for that panel. The reported conditional correction is original apparent AUC (1.000000) minus mean optimism (0.075258858), giving 0.924741142. This is an internal estimate conditional on the 491 successful panel constructions. It is not the mean AUC across held-out external patients.

Nine attempts failed to construct four genes: iterations 124, 130, 204, 218, 273, 304, 309, 328 and 329. They are reported, not silently replaced or assigned an arbitrary AUC. Mean training and original-cohort evaluation AUCs among successful attempts are 0.808194557 and 0.732935700. There are 92 training and 82 original-cohort AUCs below 0.5. No direction reversal removes them. A total of 610 distinct genes enters successful panels, and *MRPL55* recurs 30 times. These diagnostics are essential when interpreting the high conditional corrected value.

A sampling confidence interval for the conditional corrected estimator is unavailable. Monte Carlo error of the mean optimism is not such an interval. Likewise, a conventional DeLong interval of 1–1 after complete in-sample separation does not account for gene selection or establish certainty.

Independent re-fitting used R 4.3.3, limma 3.58.1, glmnet 4.1-8 and Matrix 1.6-5 on Linux, compared with source R 4.5.2, limma 3.66.0, glmnet 4.1-10 and Matrix 1.7-4 on Windows. Saved discovery folds were supplied to newly fitted models. Each bootstrap draw and fold assignment was regenerated from seed 20260915 + iteration and checked against the saved assignments. Top-200 rankings and selected panels matched all 491 successful records; all nine failure outcomes matched. The maximum coefficient difference was below 6.83 × 10^-14. The discovery frequencies, mean optimism and conditional corrected AUC were reproduced. Scripts, logs, package versions and per-replicate re-fits are supplied in S1–S4 Data. This verifies the reconstructed procedure under the tested environments, not every possible software environment.

# M5 Targeted pathway and correlation analyses

The three targeted panels are *TNF*/*NFKB1*/*RELA*/*ICAM1*, *IL6*/*CXCL8*/*CCL2*/*STAT3* and *IFNG*/*STAT1*/*IRF1*/*CXCL10*. They are four-gene panels, not full Hallmark pathways. Saved GSVA parameters are kcdf = Gaussian, minSize = maxSize = 4, tau = 1, maxDiff = TRUE and absRanking = FALSE. Source package versions are GSVA 2.4.7 and limma 3.66.0. Limma contrasts responders minus non-responders, and Benjamini–Hochberg adjustment covers all three contrasts.

Mean score differences, nominal *P* values and adjusted *P* values are: *TNF* −0.274166799, 0.231239479, 0.346859219; inflammatory −0.102720254, 0.652242507, 0.652242507; *IFNG* −0.424514357, 0.065789746, 0.197369239. These are differences in GSVA scores, not expression log-fold changes, and none establishes significance.

S3 Fig uses Spearman correlations among four-gene expression, *IFNG* score and response coded 1/0. S4 Fig plots the mean four-gene score against *IFNG*, with overall Spearman rho = 0.205217391. Both are descriptive analyses after gene selection on these same samples. They do not establish incremental prediction, independence of markers or a combined clinical model. No inferential stars or correlation *P* values are assigned.

# M6 External blood cohort

GSE191328 provides baseline vedolizumab blood data for 17 patients, including ten UC and seven Crohn’s disease cases. The source week-14 remission labels identify nine with remission and eight without; the UC subset has six/four. The four target symbols each map to one Entrez Gene identifier in the saved mapping. Source normalized TPM values are used without external gene selection or fitting. All four genes are required; the score is their mean TPM with the discovery direction retained. *TPRXL* is zero in 14 of 17 blood samples. AUCs are 0.458333333 overall and 0.500000 in UC. These endpoints and populations differ from the composite discovery setting.

# M7 E-MTAB-7845 sample selection and endpoint

The SDRF and ERP114636 run manifest identify 47 RNA-seq samples in the available study metadata. Twenty Crohn’s disease samples were excluded. The UC analysis comprises 27 samples: Inception 20 (13 responders/seven non-responders) and Validation seven (three/four). The source publication, Verstockt et al., Clin Gastroenterol Hepatol. 2020;18:1142–1151.e10, doi:10.1016/j.cgh.2019.08.030, defines UC endoscopic remission as Mayo endoscopic subscore ≤1 at week 14 of vedolizumab treatment. It describes the RNA-seq cohorts separately from GSE73661, which is an independent historical cohort in that publication. This supports study-reported independence; cross-study identifying patient records were unavailable for direct deduplication.

Older audit notes in the source archive predate the endpoint/publication check and mark endpoint timing or independence as pending. The dated clarification in S1–S4 Data supersedes those historical notes. Response labels are not retrospectively changed to improve discrimination.

# M8 RNA-seq processing and annotation

SRA Toolkit 3.0.3 downloaded and converted the relevant reads to FASTQ. The original reads were single-end, 51 bases long. Fastp 0.23.4 used the explicit adapter AGATCGGAAGAGCACACGTCTGAACTCCAGTCA, eight threads, qualified_quality_phred = 15, unqualified_percent_limit = 40, n_base_limit = 5 and length_required = 31. Per-sample JSON reports are included in S1–S4 Data.

Salmon 1.10.2 indexed GENCODE v47 transcripts together with the GRCh38 primary assembly as genomic decoys using k = 23 and --gencode. Quantification used -l A, single-end -r input, --validateMappings, --seqBias, --fldMean 200, --fldSD 80 and eight threads. Fragment-length parameters are assumptions required for single-end analysis, not empirical measurements. All 27 libraries were inferred as SR; mapping rates range from 90.0394525% to 94.5545070%. Index duplicates were handled by the default construction; retention of duplicate transcript sequences was not requested. Salmon and fastp provenance files, rather than newly regenerated raw outputs, support the revision-stage audit.

Transcript TPM was summed to gene IDs and then to the four requested symbols. Mappings are *ZNF689*, ENSG00000156853.13; *ZC3H10*, ENSG00000135482.7; *MRPL55*, ENSG00000162910.19; and *TPRXL*, ENSG00000292995.2 plus ENSG00000180438.16. The *TPRXL* primary score sums both IDs. Sensitivities using either ID alone have the same overall UC AUC (0.534090909), but this does not establish equivalence to the discovery microarray probe coverage. S1–S4 Data includes the full 78,277-row gene-ID TPM matrix, target mappings and transcript mapping.

# M9 External scoring and statistical inference

Both external projections average raw TPM for *ZNF689*, *TPRXL*, *ZC3H10* and *MRPL55*, retaining the lower-score-predicts-positive direction. No sign flip, external coefficient fit, feature selection or threshold optimization is performed. Since microarray discovery averages log2 expression, fixing gene membership does not produce a measurement-equivalent or calibrated cross-platform model. Gene abundance can dominate the raw-TPM mean. The external estimates evaluate this declared exploratory projection only.

AUC is the fraction of positive–negative sample pairs in which the positive sample has the lower score, with ties contributing one-half. DeLong variance is calculated from the positive-wise and negative-wise pair averages; normal-approximation 95% limits are bounded to [0,1]. Score-group *P* values use two-sided Mann–Whitney/Wilcoxon rank-sum inference with the asymptotic approximation and continuity correction. They are nominal numerical *P* values, not exact small-sample tests. No multiplicity adjustment is claimed for exploratory external subgroup comparisons. Subgroups overlap their parent cohort and are not pooled.

E-MTAB-7845 overall AUC is 0.534090909 (95% CI 0.292767491–0.775414327), compared with Inception 0.648351648 (0.369602144–0.927101153) and Validation 0.333333333 (0–0.823324329). The corresponding *P* values are 0.786079500, 0.302936073 and 0.595883091. Small subgroup results are imprecise, and no favorable subgroup is selected as the primary result.

Revision-stage sensitivities evaluated both panels (submitted: *ZNF689*/*TPRXL*/*ZC3H10*/*TVP23C*; rebuilt: *ZNF689*/*TPRXL*/*ZC3H10*/*MRPL55*) on the same external samples using raw gene-TPM means and mean log2(TPM+1). The logarithm compresses abundance differences while the pseudocount of one accommodates measured zeros; it does not harmonize RNA-seq with microarray measurements. *TVP23C* maps to Entrez 201158 in the blood matrix and ENSG00000175106.17 in the mucosal GENCODE table. *TPRXL* aggregation follows the primary two-ID rule. All 20 panel/scale/cohort combinations are reported, including the UC blood and source-defined mucosal subsets. Direction was fixed before these comparisons; neither labels nor direction were changed, and no favorable variant replaces the primary analysis. These are revision-stage analyses, not preregistered tests. Nominal *P* values are unadjusted and descriptive.

For all 27 mucosal UC samples, the submitted panel has raw-TPM AUC 0.636364 (95% CI 0.422271–0.850456; *P* = 0.246195) and log2(TPM+1) AUC 0.607955 (0.387545–0.828364; *P* = 0.361291). The rebuilt panel has log2(TPM+1) AUC 0.517045 (0.244115–0.789976; *P* = 0.901817). The submitted panel has blood raw-TPM AUC 0.513889 (0.214141–0.813637; *P* = 0.961627). Full results and sample-level scores are supplied in S5 Table and S1–S4 Data.

# M10 Timepoint sensitivities

The rebuilt panel is held fixed when response is reassigned using each observed timepoint. Week 12 includes 13 patients (3/10), with AUC 0.866666667, DeLong interval 0.656653–1 and *P* = 0.075927. Week 52 includes 19 (13/6), with AUC 1 and *P* = 0.000734. Excluding vdz_plac leaves 17 (12/5), with AUC 1 and *P* = 0.001874. The exact full-precision results and sample assignments are included in S1–S4 Data. The latter two conventional intervals degenerate at 1–1.

These analyses are post-selection and overlap the composite discovery cohort. They neither remove the effect of missing outcomes nor establish unbiased induction/maintenance performance. Their purpose is to show sensitivity to the reported endpoint definitions, not to validate one timepoint as the better prediction target.

# M11 Availability and scope of re-execution

S1–S4 Data include source checkpoints, sample-level tables, gene-level RNA-seq TPM, processing metadata, numerical replay code, portable figure scripts and the revision-stage independent R analysis. The latter supplies probe aggregation, discovery re-fitting, all 500 bootstrap re-fits, nested validation, external panel/scale sensitivities, logs and session information. The complete legacy probe-audit RDS is restored from the original VDZ_revision_work.zip member; the earlier loose copy was incomplete. File checksums identify the corrected input. The original VDZ_revision_work.zip is not included in the five data archives; verifying the byte-level restoration of that RDS from the named ZIP member requires the original archive, whereas the supplied restored RDS and saved outputs permit analysis replay.

Raw FASTQ, large index binaries and individual quant.sf files are not duplicated in this compact supplement. Raw sequencing is accessioned in ERP114636; supplied gene TPM and transcript mapping support the projections. Neither raw microarray CEL preprocessing, GSVA model fitting nor FASTQ-to-Salmon quantification was independently repeated in this revision. Existing GSVA scores and contrasts were numerically checked. The historical 23-sample sensitivity subset is recovered, but complete execution provenance for the later submitted model estimates remains unresolved; the independent R fits verify the documented 24-sample reconstruction.

S1 Data contains code, numerical tables, RNA-seq data and re-fitting outputs; S2 Data contains the processed-probe audit and discovery checkpoints; S3 Data contains remaining checkpoints and the reconstruction input; S4 Data contains remaining independent R inputs. S5 Data supplies historical source excerpts, comparison tables, current-panel reconciliation and verification scripts. Each of the five V8 archives is below 20 MB and opens independently. Extract all five S1–S5 Data archives into one directory. DATA_ARCHIVES.csv, SHA256SUMS.txt and Revision7/verify_payload.py identify and verify the payload. These are ordinary ZIPs, not split volumes. Full verification against the original historical workspaces additionally requires core_data.RData, VDZ_workspace.RData and VDZ_project3.zip, packaged unchanged in Historical_original_sources.zip. S5 Data contains exports and excerpts, not these complete original inputs. The three original historical source files (core_data.RData, VDZ_workspace.RData, and VDZ_project3.zip) are publicly available in the OSF project at https://osf.io/kx726/.

Nested validation evaluates the reconstructed selection procedure, whereas the final four-gene set is evaluated separately in exploratory external projections. Earlier processed-array normalization and annotation were not re-estimated within outer folds. Previously withdrawn historical permutation results do not apply to the reconstructed 24-sample analysis. Full verification against original historical workspaces requires the source files specified above.

# M12 Repeated nested validation

An additional robustness analysis used 20 repetitions of stratified four-fold outer cross-validation on the same 24 discovery patients. Outer assignments used seed 880000 + repetition. Within each outer training set, seed 890000 + 100 × repetition + fold generated 50 stratified inner fold assignments. Limma ranking, top-200 filtering, all LASSO fits and frequency-based four-gene selection were repeated using training patients only. The held-out patients did not contribute to these label-dependent steps. Earlier processed-array normalization and annotation were not re-estimated within folds. This is internal validation of the reconstructed selection procedure, not independent validation of the fixed final panel.

The mean log2 expression of the newly selected panel was scored with the fixed lower-score-predicts-response direction in each held-out fold. Since different folds can select genes with different expression scales, scores from different panels were not pooled into one ROC curve. AUC was computed within each fold; each complete repetition averaged its four AUCs using responder–non-responder pair counts as weights. The final summary is the arithmetic mean of complete-repetition estimates. Failed panel constructions were retained and were not assigned arbitrary scores or AUCs.

Seventy-eight of 80 outer fits produced four-gene panels. Repetitions 10 and 12 each contained one failed construction, leaving 18 complete repetitions. Their mean pair-weighted AUC was 0.611111, median 0.625000 and range 0.416667–0.750000. This summary is conditional on complete repetitions. The range describes sensitivity to sample splitting, not a sampling confidence interval; repetitions reuse patients and are not independent cohorts. The endpoint and candidate strategy were developed in this small source dataset, so this result does not remove all analyst-selection uncertainty. All outer assignments, held-out scores, panels and failure records are provided.

The bootstrap estimate subtracts average optimism from the apparent AUC, with the resample-derived panels evaluated in their bootstrap samples and the overlapping original cohort. Nested validation instead evaluates each panel in patients excluded from its outer training subset. Its training sets contain 17 or 19 patients. Different training and evaluation conditions, unstable selection and conditioning on successful construction limit direct comparison of the two estimates. Fig 3F and separate rows in Table 2 display both results without treating them as interchangeable.

During revision, greater interpretive weight was given to held-out nested validation and external projections than to the conditional bootstrap-corrected estimate. This interpretation was adopted during revision, not prospectively specified.

# M13 AI assistance and computational checks

ChatGPT/Codex (OpenAI) assisted with language editing, revision planning, code drafting, numerical cross-checks and figure preparation. AI-assisted outputs were checked programmatically against source metadata and saved numerical records, and the independent R fits were executed with logged versions and retained results. Independent here means separately implemented and executed calculations; it does not claim review by a separate human analyst or replace author verification. No experimental observations were generated. The authors are responsible for assessing the source information, methods, interpretations and final submission. The manuscript, relevant figure legends and reviewer response disclose this scope.

# M14 Recovered historical analysis and its relation to the revision

The recovered VDZ_workspace.RData, core_data.RData and VDZ_project3.zip contain matching historical main-analysis objects for 24 patients (14 responders/10 non-responders). The console record creates model_meta2 after baseline–outcome merging and removal of missing response labels. It then creates a separate sensitivity set using keep_idx2 <- model_meta2$patient_id != "64". The corresponding 23-sample matrix is exactly the main matrix with GSM1900320 removed (14 responders/nine non-responders). Patient 64 has recorded endoscopic Mayo 3 at both W12 and W52. Recovery establishes the operation and identity, not an objective prespecified exclusion criterion or a complete link to every later submitted result.

Historical *TVP23C* expression was the mean of probes 8005501 and 8012924, whose multi-gene mappings are documented in M3. The recovered historical selection record used the 21,176-gene matrix, scaling before 24 leave-one-out feature-selection iterations, lambda.min and a final threshold of at least five selections. *TPRXL*, *ZNF689*, *TVP23C* and *ZC3H10* appeared 16, 15, 14 and 12 times, respectively; *MRPL55* appeared three times and did not meet that threshold. These records do not establish execution of the later manuscript description of top-200 filtering, 50 repetitions and lambda.1se for the original panel. This is a discrepancy between recovered records and the submitted description; it does not establish that no later execution occurred elsewhere.

The current reconstruction applies uniform single-gene probe filtering and the distinct procedure in M3. Its saved folds and coefficient matrices reproduce ZNF689/TPRXL/ZC3H10/MRPL55 and the MRPL55–SIRPD frequency tie. Independent R fitting records reproduce these frequencies and the current bootstrap results. The annotation change, retention of patient 64 and revised selection procedure are therefore reported separately; none is treated as an isolated explanation for every historical numerical change.

Verification of the recovered historical saved data reproduced the fixed four-gene mean-score apparent AUC of 1.000 in both the 24-patient set and the patient-64-excluded subset, and the 24-patient gene-plus-*IFNG* score AUC of 0.957143. This verification recalculated saved scores and expression summaries; it did not rerun historical limma empirical-Bayes inference, LASSO selection or the complete original bootstrap. Submitted values of 0.960, 0.841 and 0.724 remain unmatched to complete execution records and are not presented as reproduced. A historical CV vector contains only 18 finite predictions and six missing values; its finite-case AUC of 0.636364 is not a complete-cohort validation estimate and is not used as revised validation evidence. Those 18 predictions reuse discovery patients and do not constitute an independent validation cohort. A matching rounded value alone would not establish the provenance of a submitted figure or estimate.

S5 Data provides source hashes, source excerpts, the 24-versus-23 sample ledger, patient-64 follow-up, probe mappings, historical frequencies and verification scope. The current-panel evidence is kept distinct from the historical records. These provenance findings improve traceability but do not establish external discrimination, biological uniqueness of the four genes or retrospective prespecification.
