# Supplementary material for: Exploratory baseline mucosal transcriptomics and vedolizumab response in ulcerative colitis
Source: PLoS One. 2026 Oct 26;21(10):e0361301. doi: 10.1371/journal.pone.0361301 (PMC13705592; doi:10.1371/journal.pone.0361301)
Supplement: Supplementary file 3 [file pone.0361301.s007.docx]

S3 Fig. Descriptive correlations within the reconstructed discovery cohort

Spearman correlations among four-gene log2 expression, the targeted IFNG-panel score and composite response (1 = responder, 0 = non-responder) in 24 samples (14/10). Cell labels show correlation coefficients. These post-selection associations are descriptive, with no significance tests or independent-validation claim. ChatGPT/Codex (OpenAI) assisted with figure preparation; figure content and numerical annotations were checked against saved analysis outputs.
