# Supplementary material for: Exploratory baseline mucosal transcriptomics and vedolizumab response in ulcerative colitis
Source: PLoS One. 2026 Oct 26;21(10):e0361301. doi: 10.1371/journal.pone.0361301 (PMC13705592; doi:10.1371/journal.pone.0361301)
Supplement: Supplementary file 4 [file pone.0361301.s008.docx]

S4 Fig. Relationship between the rebuilt four-gene score and the targeted IFNG-panel score

Each point represents one of 24 discovery samples (14 responders/10 non-responders). Spearman rho is 0.205217. This post-selection companion display does not establish incremental prediction, a combined model or external validity, and is not a co-primary model. ChatGPT/Codex (OpenAI) assisted with figure preparation; figure content and numerical annotations were checked against saved analysis outputs.
