# Supplementary material for: Exploratory baseline mucosal transcriptomics and vedolizumab response in ulcerative colitis
Source: PLoS One. 2026 Oct 26;21(10):e0361301. doi: 10.1371/journal.pone.0361301 (PMC13705592; doi:10.1371/journal.pone.0361301)
Supplement: Supplementary file 5 [file pone.0361301.s009.docx]

S5 Table. Analysis reconstruction and revision-stage sensitivity checks

Historical and reconstructed panel/annotation comparison, fixed-direction external panel/scale results, independent R re-fitting and repeated nested validation. These added sensitivities were not prospectively specified and do not replace the primary analysis.

PONE-D-26-17559 | These analyses were added during revision. They were not prospectively specified and do not replace the primary analysis.

# A. Submitted and reconstructed analyses

| **Item** | **Submitted and recovered historical analysis** | **Documented reconstruction** |
| --- | --- | --- |
| Discovery population | Submitted: 23 samples. Recovered: main 24 (14/10); exclude-patient-64 sensitivity 23 (14/9). | 24 baseline samples: 14 responders / 10 non-responders |
| Four-gene panel | *ZNF689*, *TPRXL*, *ZC3H10*, *TVP23C* | *ZNF689*, *TPRXL*, *ZC3H10*, *MRPL55* |
| *TVP23C* probe 8005501 | One of the two probes averaged for historical TVP23C expression | *TVP23B*; *TVP23C*; *TVP23C*-CDRT4: excluded by single-gene rule |
| *TVP23C* probe 8012924 | One of the two probes averaged for historical TVP23C expression | *TVP23C*; *TVP23C*-CDRT4: excluded by single-gene rule |
| Scope of verification | Main/sensitivity objects, deletion code and fixed scores recovered; submitted 0.960/0.841/0.724 execution records remain unmatched. | 24-sample processed-probe aggregation and selection independently re-executed |

Recovered records distinguish a 24-patient main analysis (14 responders/10 non-responders) from a sensitivity analysis excluding patient 64, GSM1900320 (23 patients; 14 responders/nine non-responders). Both follow-up Mayo subscores for this patient were 3. The deletion operation is recovered; an objective prespecified exclusion criterion and the link to every later submitted 23-sample estimate remain unverified. Historical TVP23C expression averaged the two mapped probes listed above. *Their* exclusion under the revised single-gene rule and subsequent re-selection are distinct from the sample-count correction. See S1 Methods M14 and S5 Data.

# B. Independent R re-fitting checks

| **Check** | **Result** |
| --- | --- |
| Probe-to-gene aggregation | 33,252 probes → 19,871 eligible single-gene probes → 18,865 genes; all 24 labels identical; maximum expression difference 0 |
| Discovery selection | Identical top-200 list and final panel; counts 41/38/36/20 of 50 fits. MRPL55 and SIRPD tied at 20; limma ranks 23 versus 55 retained MRPL55. |
| Bootstrap construction | 500 attempts; 491 successes and 9 failures, all matching saved outcomes |
| Bootstrap candidate/panel agreement | 491/491 successful top-200 lists and final panels matched; maximum coefficient difference <6.83 × 10^-14 |
| Bootstrap estimates | Mean optimism 0.075259; conditional corrected AUC 0.924741; mean training / original-cohort AUCs 0.808195 / 0.732936 |

Source: R 4.5.2, limma 3.66.0, glmnet 4.1-10, Matrix 1.7-4 (Windows). Re-fitting: R 4.3.3, limma 3.58.1, glmnet 4.1-8, Matrix 1.6-5 (Linux). The independent checks concern the reconstructed procedure; raw CEL preprocessing, GSVA fitting and FASTQ quantification were not repeated.

# C. Fixed-direction external panel and scale sensitivities

Submitted panel (S): *ZNF689*/*TPRXL*/*ZC3H10*/*TVP23C*. Rebuilt panel (R): *ZNF689*/*TPRXL*/*ZC3H10*/*MRPL55*. TPM = arithmetic mean of raw gene TPM; log = mean log2(TPM+1). Lower scores predict response throughout.

| **Cohort** | **Panel** | **Scale** | **n R/NR** | **AUC** | **95% CI** | ***P*** |
| --- | --- | --- | --- | --- | --- | --- |
| Blood, all IBD | S | TPM | 9/8 | 0.5139 | 0.2141–0.8136 | 0.961627 |
| Blood, all IBD | S | log | 9/8 | 0.5278 | 0.2305–0.8251 | 0.885234 |
| Blood, all IBD | R | TPM | 9/8 | 0.4583 | 0.1619–0.7548 | 0.809894 |
| Blood, all IBD | R | log | 9/8 | 0.4444 | 0.1517–0.7372 | 0.736277 |
| Blood, UC | S | TPM | 6/4 | 0.5417 | 0.0674–1.0000 | 0.915106 |
| Blood, UC | S | log | 6/4 | 0.5417 | 0.0674–1.0000 | 0.915106 |
| Blood, UC | R | TPM | 6/4 | 0.5000 | 0.0658–0.9342 | 1.000000 |
| Blood, UC | R | log | 6/4 | 0.4583 | 0.0272–0.8894 | 0.915106 |
| Mucosa, all UC | S | TPM | 16/11 | 0.6364 | 0.4223–0.8505 | 0.246195 |
| Mucosa, all UC | S | log | 16/11 | 0.6080 | 0.3875–0.8284 | 0.361291 |
| Mucosa, all UC | R | TPM | 16/11 | 0.5341 | 0.2928–0.7754 | 0.786079 |
| Mucosa, all UC | R | log | 16/11 | 0.5170 | 0.2441–0.7900 | 0.901817 |
| Mucosa, inception | S | TPM | 13/7 | 0.6264 | 0.3729–0.8798 | 0.383387 |
| Mucosa, inception | S | log | 13/7 | 0.6374 | 0.3870–0.8878 | 0.341648 |
| Mucosa, inception | R | TPM | 13/7 | 0.6484 | 0.3696–0.9271 | 0.302936 |
| Mucosa, inception | R | log | 13/7 | 0.6484 | 0.3168–0.9799 | 0.302936 |
| Mucosa, validation | S | TPM | 3/4 | 0.6667 | 0.1677–1.0000 | 0.595883 |
| Mucosa, validation | S | log | 3/4 | 0.5000 | 0.0000–1.0000 | 1.000000 |
| Mucosa, validation | R | TPM | 3/4 | 0.3333 | 0.0000–0.8233 | 0.595883 |
| Mucosa, validation | R | log | 3/4 | 0.2500 | 0.0000–0.7400 | 0.376759 |

CIs use the DeLong variance and a normal approximation bounded to 0–1. *P* values are two-sided, asymptotic, continuity-corrected Wilcoxon tests; the 20 exploratory comparisons are unadjusted. Cohort subsets overlap and are not independent studies. All CIs include 0.5; these results do not establish discrimination or equivalence to chance.

*TVP23C*: blood Entrez 201158; mucosa ENSG00000175106.17. *TPRXL* uses the primary two-ID sum. The logarithmic sensitivity compresses abundance differences and retains measured zeros; it does not calibrate or harmonize RNA-seq and microarray expression. No panel, scale, subgroup or score direction was chosen to improve the primary result.

# D. Repeated nested validation

All 24 discovery patients entered 20 repetitions of stratified four-fold outer validation. Limma ranking, top-200 filtering and 50 LASSO selections were repeated within each outer training set. Pre-existing array normalization and annotation were not re-estimated within folds. Each newly selected panel was evaluated only in its held-out fold, with fixed direction.

Summary: 78/80 outer fits succeeded; 18 complete repetitions had mean pair-weighted AUC 0.611111, median 0.625000 and range 0.416667–0.750000. AUCs within each repetition were weighted by responder–non-responder pair counts. Scores from different selected panels were not pooled.

| **Repetition** | **Successful outer folds** | **Pair-weighted held-out AUC** |
| --- | --- | --- |
| 1 | 4/4 | 0.694444 |
| 2 | 4/4 | 0.555556 |
| 3 | 4/4 | 0.638889 |
| 4 | 4/4 | 0.750000 |
| 5 | 4/4 | 0.666667 |
| 6 | 4/4 | 0.722222 |
| 7 | 4/4 | 0.527778 |
| 8 | 4/4 | 0.555556 |
| 9 | 4/4 | 0.638889 |
| 10 | 3/4 | Not estimable: one construction failed |
| 11 | 4/4 | 0.444444 |
| 12 | 3/4 | Not estimable: one construction failed |
| 13 | 4/4 | 0.472222 |
| 14 | 4/4 | 0.583333 |
| 15 | 4/4 | 0.416667 |
| 16 | 4/4 | 0.583333 |
| 17 | 4/4 | 0.694444 |
| 18 | 4/4 | 0.611111 |
| 19 | 4/4 | 0.694444 |
| 20 | 4/4 | 0.750000 |

Failures in repetitions 10 and 12 were retained without arbitrary replacement scores. The summary is conditional on the 18 complete repetitions. The range reflects sensitivity to splitting, not a confidence interval; repetitions reuse the same patients. This is internal validation of the selection procedure, not external validation of the final fixed panel. Complete assignments, panels, held-out scores and failure records are included in S1–S4 Data; see S1 Methods M12. The held-out estimate is also displayed in Fig 3F and a separate Table 2 row; its training and evaluation conditions differ from those of bootstrap optimism correction.
