# Supplementary material for: Exploratory baseline mucosal transcriptomics and vedolizumab response in ulcerative colitis
Source: PLoS One. 2026 Oct 26;21(10):e0361301. doi: 10.1371/journal.pone.0361301 (PMC13705592; doi:10.1371/journal.pone.0361301)
Supplement: Supplementary file 6 [file pone.0361301.s012.docx]

Response to the Academic Editor and Reviewer

PONE-D-26-17559

Revised title: Exploratory baseline mucosal transcriptomics and vedolizumab response in ulcerative colitis

Dear Academic Editor and Reviewer,

Thank you for identifying the limitations in validation, statistical interpretation and data transparency. We have revised the manuscript and supplied the supporting analyses. Comments below are restated for clarity, followed by the specific changes and their locations. Locations refer to the revised clean manuscript.

An important correction applies across the responses. The revised discovery analysis includes 24 baseline GSE73661 samples and a panel containing *MRPL55* rather than *TVP23C*. Newly recovered source records establish that 24 was the historical post-merge main cohort; a separately named sensitivity excluded patient 64, giving 23 samples. The exclusion command and original *TVP23C* probe construction are now recovered. A preset technical justification for exclusion and the provenance of all later submitted 23-sample model estimates remain unverified. Annotation and selection changed during revision, so the revised estimates replace rather than reproduce the submitted estimates. Current *MRPL55* selection is supported by saved fits and independent R re-fitting. The findings remain post-selection separation, unstable feature selection and no confirmed external discrimination.

The recovered selection records differ from the procedure described in the submitted Methods. The historical 18-case CV calculation reuses discovery patients and is not independent validation.

# Journal requirement 1

Comment: Ensure that the manuscript meets PLOS ONE style requirements, including file naming.

Response: We have standardized statistical notation, table presentation and Vancouver-style references, and supplied continuous line numbering and page numbering. The clean manuscript retains figure captions and contains no embedded figures. Separate Fig 1–5 files use Times-family text at 8–12 points in the visible artwork and meet the stated dimensions and resolution. Fig 1 uses lowercase limma consistently and the mathematical symbol ≤; its PDF contains only Times resources, and the TIFF is the submission file. File names, captions and in-text citations have been synchronized. We supply a clean manuscript, a Word tracked-changes copy relative to the initial submission, and separate TIFF figures.

Location: Entire manuscript and submission files.

# Journal requirement 2

Comment: Upload the Supporting Information Figures and Table referred to in the manuscript.

Response: We have replaced the previous placeholders with the actual 24-sample audit (S2 Table), the descriptive correlation matrix (S3 Fig) and the joint score display (S4 Fig). These report the documented reconstructed analysis. We also provide S1 Methods and S1–S4 Data with sample-level numerical data, saved analysis objects, bootstrap records and replay/figure code. S4 remains descriptive and is not a co-primary model. S5 Table now supplies the panel/annotation comparison, all external panel/scale sensitivities, independent re-fitting checks and repeated nested validation. The data are supplied as five independently readable ZIP archives, each below 20 MB, with preserved relative paths and a common archive manifest and checksum list. S5 Data adds the recovered historical sample and probe records, source code, checksums and numerical checks, with source-input dependencies stated. S2 Table now distinguishes historical main and exclusion-sensitivity membership from current inclusion. Full verification against the original historical workspaces also requires core_data.RData, VDZ_workspace.RData and VDZ_project3.zip. These are packaged unchanged in Historical_original_sources.zip and listed separately in the submission map. The three original historical source files (core_data.RData, VDZ_workspace.RData, and VDZ_project3.zip) are available to editors and reviewers in a private OSF project through the view-only link https://osf.io/kx726/overview?view_only=4787a4e28d264cee953ff375247436e0. The project is private for peer review; public access to these files and a permanent public repository link will be provided before publication.

Location: Supporting information captions; S1 Methods; S1–S4 Data; S2 Table; S3 and S4 Figs; S5 Data; S2 Table historical-inclusion sheet.

# Academic Editor major comment 1

Comment: E-MTAB-7845 is identified as an independent same-tissue baseline vedolizumab cohort, but no projection is performed. This is the largest limitation. Perform a locked projection if feasible, or state the limitation and soften the title and abstract; include exploratory or discovery-phase.

Response: We agree that the absence of a same-tissue analysis required more than a wording change. We now report an exploratory projection into all 27 baseline UC mucosal samples from E-MTAB-7845 (16 with endoscopic remission and 11 without). The source publication defines UC remission as Mayo endoscopic subscore ≤1 at week 14 and describes the RNA-seq cohorts separately from the historical GSE73661 cohort. Independence is therefore source-reported at study level; we do not claim direct cross-study patient-identifier deduplication. The overall AUC is 0.534091 (95% CI 0.292767–0.775414; *P* = 0.786079). The Inception and Validation subcohorts are reported without choosing the better-performing subset.

A necessary reconstruction issue is now explicit. Recovered code and saved objects identify a 24-sample post-merge main analysis (14/10) and a separate sensitivity excluding patient 64 (23 samples; 14/9). The patient has observed week-12 and week-52 Mayo subscores of 3, so missing outcome is not a reason for exclusion. No preset technical exclusion criterion was recovered. The revised discovery analysis includes all 24 recoverable baseline samples and yields *ZNF689*, *TPRXL*, *ZC3H10* and *MRPL55*, rather than the submitted *TVP23C*-containing panel. The new analysis replaces the submitted numerical results; it does not reproduce or validate the original panel. Gene membership and the lower-score-predicts-response direction were fixed for the external projections, but mean raw TPM is not equivalent to the discovery mean log2-expression score. We therefore describe the external work as an exploratory cross-platform projection, not a calibrated locked-model validation. It did not establish external discrimination. The title now begins with “Exploratory,” and the abstract and discussion no longer claim tissue specificity.

We additionally evaluated the originally submitted *TVP23C*-containing panel on the same external cohorts. Its mucosal UC AUC is 0.636364 (95% CI 0.422271–0.850456; *P* = 0.246195), and its blood AUC is 0.513889 (0.214141–0.813637; *P* = 0.961627). Mean log2(TPM+1) sensitivities give mucosal AUCs of 0.607955 for the submitted panel and 0.517045 for the rebuilt panel. All cohort, panel and scale combinations are reported in S5 Table without selecting a favorable result. These checks address the original panel directly but remain exploratory and do not establish external predictive validity.

The annotation audit now supplies a concrete reason *TVP23C* cannot enter the reconstructed candidate set: both associated probes have multiple symbols in the saved mapping and fail the single-gene rule. The original *TVP23C* expression is now traced to the mean of probes 8005501 and 8012924; both fail the uniform single-gene filter used during revision. Historical selection used all 21,176 genes, 24 leave-one-out selections, lambda.min and a frequency threshold of five. Its saved frequencies were *TPRXL* 16, *ZNF689* 15, *TVP23C* 14 and *ZC3H10* 12; *MRPL55* occurred three times and did not qualify. The reconstruction instead uses 18,865 unambiguously annotated genes, limma top-200 ranking, 50 lambda.1se fits and a four-gene frequency rule. Current *MRPL55* selection is supported by 20/50 fits, tied with *SIRPD* and resolved by the saved limma ordering (23rd versus 55th). Independent R re-fitting reproduces this panel and frequencies. These are revision-stage choices, not recovered original prespecification. We therefore do not attribute the gene change or all numerical differences to one added patient. The recovered exclusion sensitivity does not establish a complete link to every later submitted 23-sample estimate. Retaining patient 64 addresses the absence of a recovered technical exclusion justification; the uniform single-gene filter addresses ambiguous attribution. Implementing the selection workflow described in the submitted Methods is separate from reproducing the recovered historical algorithm. Agreement with the submitted Methods does not explain the historical discrepancy or establish that the revised procedure is superior. The recovered materials do not establish the complete execution path for the submitted AUCs of 0.960, 0.841 and 0.724, or the reasons and timing of the saved analytic choices.

Location: Title; Abstract; Results, “Exploratory projections in external blood and mucosal cohorts”; Methods, “Public datasets and cohort construction” and “Exploratory cross-platform projections”; Fig 5; Tables 1–2; S1 Methods; S5 Table; S1 Methods M3, M9 and M11. S1 Methods M14; S2 Table historical inclusion; S5 Data.

# Academic Editor major comment 2

Comment: Full-pipeline bootstrap reduces apparent AUC from 0.841 to optimism-corrected AUC 0.724 and shows unstable gene selection. Treat the four-gene panel as provisional and emphasize the mucosal signal rather than its exact composition.

Response: We have removed definitive-signature language and explicitly disclose that reconstruction changes the cohort, annotation and selected panel. Consequently, the previous 0.841/0.724 estimates are superseded; the new values are not presented as an improvement over them. In the reconstructed analysis, apparent AUC is 1.000000, and 491 of 500 full-pipeline bootstrap attempts construct a four-gene panel. Nine attempts fail because fewer than four genes are selected. Mean optimism is 0.075259, yielding an internal corrected AUC of 0.924741 conditional on successful construction. No confidence interval for that corrected estimator is available, and none has been invented.

We report the less favorable resampling evidence alongside this high conditional point estimate: mean bootstrap-training and original-cohort evaluation AUCs are 0.808195 and 0.732936; 610 distinct genes enter panels; and *MRPL55* appears in only 30 of 491 successful panels. AUCs below 0.5 and negative optimism are retained without direction reversal. Fig 3 and Table 2 now expose selection instability and failures. The discussion restricts the supported observation to post-selection separation within the discovery cohort and acknowledges that independent mucosal discrimination was not established.

We have now independently re-executed the 24-sample reconstruction in R. Probe aggregation reproduces the saved 18,865-gene matrix exactly. Discovery frequencies match, and all 500 bootstrap construction outcomes are reproduced, with identical top-200 candidate sets and panels for all 491 successes. The maximum coefficient difference across bootstrap re-fits is below 6.83 × 10^-14 despite documented R/package-version differences. This goes beyond checking stored AUC arithmetic.

We also added 20 repetitions of stratified four-fold outer validation, repeating all label-dependent ranking and 50 LASSO fits inside each training subset. Seventy-eight of 80 fits succeed; 18 complete repetitions give mean within-fold, pair-weighted AUC 0.611111 (range 0.416667–0.750000). We report the two failed constructions and do not interpret the repeat range as a confidence interval. This evaluates the reconstructed selection procedure rather than independently validating the fixed final four-gene panel; the mean is conditional on the 18 complete repetitions. Earlier processed-array normalization and annotation were not re-estimated within folds. The much lower held-out performance reinforces the need to avoid reading the conditional 0.924741 estimate as a clinically validated performance level. Historical main and exclusion-sensitivity objects are now recovered. They reproduce the historical four-gene mean-score AUC of 1.000 in both 24 and 23 samples, but do not reproduce the submitted 0.960, 0.841 or 0.724 estimates. A separate saved historical cross-validation vector contains only 18 finite scores and six missing values; its complete-case AUC is 0.636364 and cannot be presented as full-cohort validation. These historical calculations are documented separately and do not replace the current nested validation.

Fig 1 now shows the outer validation step, Fig 3F displays the held-out estimate alongside the conditional bootstrap result, and Table 2 reports nested validation in a separate row. Methods now explain that bootstrap evaluation against the original cohort includes patients used in panel construction, whereas each outer test fold is excluded from its panel derivation. The two estimates use different training and evaluation conditions and are not interchangeable. We now explicitly give greater interpretive weight to the held-out nested estimate and external projections; the conditional bootstrap correction is retained as an internal diagnostic. This is a revision-stage interpretation of the existing analyses, not a prospectively specified analysis hierarchy. The historical finite-case CV estimate comes from the same discovery cohort and is not an independent line of validation. We do not infer absence of an association from nonsignificant results or wide confidence intervals.

Location: Abstract; Results, “A rebuilt four-gene panel shows in-sample separation with unstable composition”; Discussion; Methods, “Internal evaluation and resampling”; Fig 3; Table 2; S1–S4 Data; S5 Table; S1 Methods M3–M4, M11–M12; independent R code and logs in S1–S4 Data; S1 Methods M14; S5 Data.

# Academic Editor major comment 3

Comment: The pragmatic Mayo 0–1 response definition at weeks 12 and 52 groups induction and maintenance outcomes. Provide separate timepoint sensitivities if possible, or explain why not. Cite VERDICT for outcome context.

Response: We now define the composite outcome precisely: Mayo endoscopic subscore 0–1 at either observed week-12 or week-52 assessment, with no imputation of missing assessments. Separate observed-timepoint analyses are supplied. At week 12, n = 13 (3 responders/10 non-responders), AUC = 0.866667 (95% CI 0.656653–1.000000; *P* = 0.075927). At week 52, n = 19 (13/6), AUC = 1.000000 (*P* = 0.000734). Excluding the source vdz_plac category leaves n = 17 (12/5), AUC = 1.000000 (*P* = 0.001874). Sample membership, missing outcomes and treatment codes are provided in S2 Table.

These subsets overlap and use genes selected with the composite outcome; they are not independent validation cohorts. Degenerate 1–1 intervals after complete separation are identified as uninformative about selection uncertainty. We discuss missing-outcome bias, treatment changes and the selection dependence of the week-52 result. VERDICT is cited for the relevance of endoscopic treatment targets, while explicitly avoiding a claim that its protocol validates our W12/W52 pooling.

Location: Results, “Biological context and study boundaries”; Discussion; Methods, “Expression processing and harmonisation” and “Interpretive analyses”; Table 2; S2 Table; S1 Methods.

# Academic Editor major comment 4

Comment: The four genes have weak direct gut-immunology links; label them mucosal state markers rather than mechanistic mediators.

Response: We retain the interpretation as candidate mucosal state markers and remove causal or trafficking-specific implications. The reconstructed panel contains *MRPL55* rather than *TVP23C*. We therefore withdraw the previous indirect *TVP23B* barrier argument, describe *MRPL55* only in terms of mitochondrial ribosomal annotation, and qualify *ZC3H10*, *TPRXL* and *ZNF689* interpretations using the relevant literature. The original GSE234736 sorted-cell panel and dependent text are removed because the expression matrix and sample annotations could not be sufficiently traced. No replacement cell-lineage claim is made. The new probe audit identifies the two ambiguous *TVP23C* mappings and their exclusion; the original combination is retained as a separately reported external sensitivity.

Location: Discussion, gene-interpretation paragraph; Methods, “Interpretive analyses”; revised Fig 5; reference list.

# Academic Editor major comment 5

Comment: Engage with recent precision-medicine literature (PMID 39944752), explain baseline mucosal profiling, and discuss complementary deep-healing approaches (PMID 39944363).

Response: We have added Little et al. on precision medicine to the introduction and discussion, and Maeda et al. on advanced endoscopic imaging and deep healing to the discussion. The added text positions mucosal transcriptomics as one possible component of molecular, endoscopic and clinical assessment. It does not claim that an exploratory four-gene score can substitute for these assessments or has demonstrated incremental clinical utility.

Location: Introduction, baseline-profiling rationale; Discussion, precision-medicine paragraph; references to Little et al. and Maeda et al.

# Academic Editor major comment 6

Comment: Discuss VERDICT (PMID 38336367) for outcome definitions and MOVE-IT (PMID 41453773) for biomarkers and precision dosing.

Response: Both protocols are now cited and their roles distinguished. The manuscript describes their trial designs without treating the cited protocols as completed comparative efficacy results. VERDICT provides context for comparing UC treatment targets and the use of endoscopic outcomes, not evidence that combining our induction and maintenance observations is valid. MOVE-IT provides context for model-informed vedolizumab dosing. We explicitly state that the present transcriptomic findings do not justify dose selection or treatment changes. Neither protocol is represented as validation of this gene panel.

Location: Discussion, precision-medicine paragraph and concluding paragraph; references to Jairath et al. and Frimor et al.

# Academic Editor minor comment 1

Comment: The title should include exploratory or discovery-phase.

Response: The title is now “Exploratory baseline mucosal transcriptomics and vedolizumab response in ulcerative colitis.” The short title has also been revised, and the tissue-restricted wording has been removed.

Location: Title page.

# Academic Editor minor comment 2

Comment: State in the abstract that the four-gene panel is exploratory and the interferon-gamma finding is nominal.

Response: The abstract labels the panel exploratory and reports the *IFNG* result as nonsignificant: mean GSVA-score difference −0.425, nominal *P* = 0.065790 and adjusted *P* = 0.197369. It also includes the negative external mucosal projection and states that external predictive validity and tissue specificity are not established. The abstract now also reports the lower nested-validation AUC, explicitly identifying this as evaluation of the selection procedure rather than independent validation of the final gene set. The abstract now prioritizes held-out nested validation and the external projections; the conditional bootstrap-corrected estimate remains reported in the Results and Table 2.

Location: Abstract.

# Academic Editor minor comment 3

Comment: Figure legends should include exact n per group, AUC confidence intervals and *P* values.

Response: We have supplied exact group sizes and the applicable AUC intervals and numerical *P* values in the legends. Fig 5 includes the overall cohorts and overlapping subgroups. Fig 4 gives nominal and BH-adjusted *P* values. Descriptive PCA/clustering and post-selection correlation displays are identified as such, rather than assigning them artificial significance tests. No inferential post-selection interval is claimed for complete apparent discovery separation, and no confidence interval is available for the conditional corrected AUC; these exceptions are stated explicitly. Fig 3F reports the nested-validation mean, complete-repetition and successful-fit denominators, and the range across splits; the range is explicitly not a confidence interval.

Location: Fig 1–5 legends; S3–S4 legends; Table 2 note.

# Academic Editor minor comment 4

Comment: Table 2 should clearly label the optimism-corrected AUC and state that it is not externally validated.

Response: Table 2 explicitly labels 0.924741 as the conditional optimism-corrected internal AUC from 491 successful bootstrap attempts. The table states that this estimate is not externally validated and has no available confidence interval. External mucosal and blood AUCs are listed separately to prevent confusion between internal correction and external evaluation. A separate row reports the mean pair-weighted held-out AUC of 0.611111 from 18 complete nested-validation repetitions, together with the 78/80 successful outer fits and the range across splits. The note distinguishes this range from a confidence interval and distinguishes the training and evaluation conditions of bootstrap correction and nested validation. S5 Table supplies the complete construction and re-fitting audit.

Location: Table 2 and its note.

# Academic Editor minor comment 5

Comment: Supplementary Figure S4 should remain explicitly non-co-primary.

Response: S4 Fig is now the descriptive relationship between the rebuilt four-gene score and the targeted *IFNG* score. It is explicitly non-co-primary, post-selection and not evidence of incremental prediction or an externally validated combined model. The previous combined-model implication is removed.

Location: Results; Methods, “Internal evaluation and resampling”; S4 Fig and caption.

# Academic Editor minor comment 6

Comment: Confirm that AI was not used for data analysis or scientific decision-making.

Response: The earlier language-only statement was too narrow and has been corrected. Large language model assistance supported manuscript preparation. It did not generate source observations or independently determine scientific conclusions. The authors remain responsible for verifying the data, analyses, figures and interpretations and for approving the final submission.

Location: Methods, “Ethics statement and AI use”.

# Reviewer 1 general assessment

Comment: The reviewer rated technical soundness as partly met, statistical rigor and data availability as not met, and English presentation as satisfactory.

Response: We have focused the revision on the substantive issues rather than stylistic rewriting. The manuscript now distinguishes the reconstructed analysis from the unreproduced submitted results; reports failed resamples, conditional correction and post-selection limitations; adds independent mucosal projection and timepoint sensitivities; and supplies numerical source data and actual supplementary figures. The claims are reduced to match these results. The historical 24-sample main analysis and explicit patient-64 exclusion sensitivity are now recovered. The objective exclusion justification and provenance of the later submitted 23-sample model estimates remain unresolved; independent re-execution verifies the documented revision-stage reconstruction. The further revision supplies executed independent limma/glmnet fits, the original-panel external analyses and repeated nested validation; raw-array preprocessing was not rerun, and the remaining historical-provenance limitations are stated precisely.

Location: Revised Results and Methods; Data availability statement; S1 Methods and S1–S4 Data; S5 Data.

# Reviewer 1 comment 1

Comment: Why was an exploratory sensitivity analysis in E-MTAB-7845 not performed? Clearly labeled exploratory analysis could inform reproducibility of the mucosal signal.

Response: We agree and have now performed the exploratory same-tissue projection using all 27 UC samples. The overall AUC is 0.534091 (95% CI 0.292767–0.775414; *P* = 0.786079). We also report both source subcohorts and the *TPRXL* annotation sensitivity, without reversing direction, refitting coefficients or selecting the better result. Both individual *TPRXL* Ensembl-ID scoring variants yield the same overall UC AUC as the primary symbol-summed score.

The primary projection applies to the rebuilt panel under the declared revision-stage annotation and selection procedure. Recovered historical code establishes a different derivation, but does not verify all submitted 23-sample model estimates. The separate projection of the submitted *TVP23C*-containing panel is also exploratory. Raw TPM averaging differs from the discovery log2 score and was not a prospectively harmonized calibration rule. We therefore retain the exploratory label and conclude that independent mucosal discrimination was not confirmed, while recognizing the wide confidence interval.

To address the originally submitted combination as well, we added its fixed-direction projection alongside the rebuilt panel. Its overall mucosal AUC is 0.636364 with raw TPM and 0.607955 with log2(TPM+1), with both intervals including 0.5. The rebuilt panel remains near chance under both scales. All 20 panel/scale/cohort combinations are reported in S5 Table; none replaces the primary analysis on the basis of performance. These are revision-stage sensitivity analyses; they are not presented as prospectively specified tests.

Location: Results and Methods, external projections; Fig 5; Table 2; S1 Methods and S1–S4 Data; S5 Table and S1 Methods M9.

# Reviewer 1 comment 2

Comment: Tissue-restricted is too strong: the mucosal and blood cohorts differ in platform, cohort characteristics and response definitions, so poor blood performance cannot be specifically attributed to tissue.

Response: We agree. Tissue-restricted/tissue-confined conclusions are removed from the title, abstract and interpretation. We explicitly discuss simultaneous differences in tissue, platform, case mix and endpoint definitions. The blood UC-only sensitivity remains uninformative (AUC 0.500000; 95% CI 0.065816–0.934184), and the independent mucosal result does not confirm discrimination. Low *TPRXL* detectability and TPM-scale gene weighting are acknowledged. The baseline Mayo endoscopic subscore range of 2–3 is retained as clinical description, without the previous claim that it eliminates severity confounding. Differences in measurement scale and endpoint timing prevent these projections from constituting same-scale validation of a calibrated model; their nonsignificant results do not prove that no association exists.

Location: Title; Abstract; Results, “Biological context and study boundaries”; Discussion, external-projection and limitations paragraphs.

# Reviewer 1 comment 3

Comment: Standardize statistical notation and tables, particularly italic *P* values.

Response: Statistical *P* is italicized throughout the revised manuscript and response document. Tables use consistent denominators, AUC/CI formatting and explicit analysis roles. We distinguish nominal from BH-adjusted *P* values and numerical reporting from the statistical meaning of an exact test: Wilcoxon results here use an asymptotic, continuity-corrected procedure. Table 2 separates apparent discrimination, conditional internal correction, repeated nested validation, independent projections and overlapping endpoint sensitivities. Table 1 now explicitly distinguishes the Mayo endoscopic subscore from a total Mayo score.

Location: Tables 1–2, figure legends, statistical methods and statistical notation throughout.

# Reviewer 1 comment 4

Comment: The interferon-gamma result did not reach conventional significance (*P* = 0.069) and should be exploratory rather than strong biological or mechanistic evidence.

Response: We have removed the implication of demonstrated interferon-gamma dysfunction or mechanistic confirmation. In the reconstructed 24-sample analysis, the corresponding nominal *P* value is 0.065790, with BH-adjusted *P* = 0.197369 across the three targeted panels. The effect is a GSVA-score difference of −0.424514, not expression logFC. The abstract, Results, Discussion and Fig 4 all state that the result is nonsignificant and exploratory. S3 and S4 remain descriptive and cannot validate the selected genes or establish a combined predictive model.

Location: Abstract; Results, “Exploratory inflammatory pathway context”; Discussion; Fig 4; S3–S4.

We hope that these changes address the requested analyses while making the remaining uncertainty explicit. The revised conclusions are restricted to post-selection in-sample separation, unstable gene selection and the absence of confirmed external discrimination.

Sincerely, The authors
