# Supplementary material for: Exploratory baseline mucosal transcriptomics and vedolizumab response in ulcerative colitis
Source: PLoS One. 2026 Oct 26;21(10):e0361301. doi: 10.1371/journal.pone.0361301 (PMC13705592; doi:10.1371/journal.pone.0361301)
Supplement: Peer review history

# Peer review: Decision Letter 0

**DOI:** 10.1371/journal.pone.0361301.r001

**Authors:** Lanlan Chen

10 Sep 2026

Dear Dr. Wang,

Thank you for submitting your manuscript to PLOS One. After careful consideration, we feel that it has merit but does not fully meet PLOS One’s publication criteria as it currently stands. Therefore, we invite you to submit a revised version of the manuscript that addresses the points raised during the review process.

Please submit your revised manuscript by Oct 25 2026 11:59PM. If you will need more time than this to complete your revisions, please reply to this message or contact the journal office at plosone@plos.org. When you're ready to submit your revision, log on to https://www.editorialmanager.com/pone/ and select the 'Submissions Needing Revision' folder to locate your manuscript file.

- A letter that responds to each point raised by the academic editor and reviewer(s). You should upload this letter as a separate file labeled 'Response to Reviewers'.
- A marked-up copy of your manuscript that highlights changes made to the original version. You should upload this as a separate file labeled 'Revised Manuscript with Track Changes'.
- An unmarked version of your revised paper without tracked changes. You should upload this as a separate file labeled 'Manuscript'.

If you would like to make changes to your financial disclosure, please include your updated statement in your cover letter. Guidelines for resubmitting your figure files are available below the reviewer comments at the end of this letter.

If applicable, we recommend that you deposit your laboratory protocols in protocols.io to enhance the reproducibility of your results. Protocols.io assigns your protocol its own identifier (DOI) so that it can be cited independently in the future. For instructions see: https://journals.plos.org/plosone/s/submission-guidelines#loc-laboratory-protocols. Additionally, PLOS One offers an option for publishing peer-reviewed Lab Protocol articles, which describe protocols hosted on protocols.io. Read more information on sharing protocols at https://plos.org/protocols?utm_medium=editorial-email&utm_source=authorletters&utm_campaign=protocols.

As the corresponding author, your ORCID iD is verified in the submission system and will appear in the published article. PLOS supports the use of ORCID, and we encourage all coauthors to register for an ORCID iD and use it as well. Please encourage your coauthors to verify their ORCID iD within the submission system before final acceptance, as unverified ORCID iDs will not appear in the published article. *Only*  the individual author can complete the verification step; PLOS staff *cannot*  verify ORCID iDs on behalf of authors.

We look forward to receiving your revised manuscript.

Kind regards,

Lanlan Chen

Academic Editor

PLOS One

Journal requirements:

When submitting your revision, we need you to address these additional requirements.

1. Please ensure that your manuscript meets PLOS ONE's style requirements, including those for file naming. The PLOS ONE style templates can be found at

https://journals.plos.org/plosone/s/file?id=wjVg/PLOSOne_formatting_sample_main_body.pdf and

https://journals.plos.org/plosone/s/file?id=ba62/PLOSOne_formatting_sample_title_authors_affiliations.pdf

2. Please upload a copy of Supporting Information Figures and Table which you refer to in your text on page 11.

If the reviewer comments include a recommendation to cite specific previously published works, please review and evaluate these publications to determine whether they are relevant and should be cited. There is no requirement to cite these works unless the editor has indicated otherwise.

Additional Editor Comments:

The topic is clinically important, and the study has several strengths: it uses publicly deposited transcriptomic data, applies a locked cross-tissue blood challenge, reports full-pipeline bootstrap resampling, and is commendably transparent about the exploratory nature of the findings. However, before the manuscript can be considered further, several major issues need to be addressed, particularly regarding the strength of the claims, the lack of independent same-tissue validation, and the integration of recent literature.

Major:

1. The manuscript identifies E-MTAB-7845 as an independent same-tissue baseline vedolizumab cohort but does not perform the projection. This is the single largest limitation. If a locked projection is not feasible, the authors must state this explicitly and soften the title and abstract accordingly. The phrase “identify a tissue-restricted signal” in the title currently overstates the strength of the evidence. I recommend revising the title to include “exploratory” or “discovery-phase,” e.g., “Exploratory baseline mucosal transcriptomics reveal a tissue-restricted signal associated with vedolizumab response in ulcerative colitis.”

2. The full-pipeline bootstrap reduced the apparent AUC from 0.841 to an optimism-corrected AUC of 0.724 and showed notable variability in gene selection. This means the four-gene panel (TPRXL, ZNF689, TVP23C, ZC3H10) is provisional. The authors should avoid presenting it as a fixed signature and instead describe it as an exploratory panel. The Discussion should explicitly state that the more robust finding is the presence of a mucosal signal, not the exact four-gene composition.

3. The pragmatic response criteria (Mayo 0–1 at weeks 12 and 52) group induction and maintenance outcomes. This may introduce heterogeneity. If possible, provide sensitivity analyses for week-12 and week-52 responders separately, or at least explain why this was not feasible. The VERDICT trial protocol (see recommended literature below) provides useful context for treatment targets in UC and should be cited when justifying the outcome definition.

4. The four genes have weak direct gut-immunology links. TVP23C is only indirectly connected to epithelial barrier biology via TVP23B; ZC3H10 is tied to mitochondrial/metabolic regulation; TPRXL and ZNF689 are largely markers of broader transcriptional states. The authors should clearly label these as mucosal state markers rather than mechanistic mediators.

5.The Introduction and Discussion would benefit from engagement with recent literature on precision medicine, treatment targets, and model-informed dosing in IBD. The authors should justify the focus on baseline mucosal transcriptomics in the introduction and position the four-gene panel within the broader precision medicine landscape in the discussion (PMID: 39944752). While not transcriptomic, the complementary precision medicine approaches and the importance of deep healing as a treatment target should be highlighted (PMID: 39944363), acknowledging that molecular biomarkers will likely be used alongside imaging and clinical data.

6. This VERDICT trial protocol is directly relevant to outcome definitions (PMID: 38336367). The authors should refer to it when discussing the choice of Mayo endoscopic subscores and the grouping of week-12 and week-52 outcomes (PMID: 38336367). The MOVE-IT protocol underscores the clinical need for biomarkers that can guide vedolizumab use (PMID: 41453773). Please discuss it to support the argument that tissue-restricted signals may eventually inform precision dosing (PMID: 41453773).

Minor

- The title should include “exploratory” or “discovery-phase.”

- The abstract should state that the four-gene panel is exploratory and that the interferon-gamma finding is nominal.

- Figure legends should include exact n per group, AUC confidence intervals, and P values.

- Table 2 should clearly label the optimism-corrected AUC and note that it is not externally validated.

- Supplementary Figure S4 should remain explicitly non-co-primary, as currently stated.

- The AI use statement is appropriate, but please confirm that no AI was used for data analysis or scientific decision-making.

[Note: HTML markup is below. Please do not edit.]

Reviewers' comments:

Reviewer's Responses to Questions

**Comments to the Author**

1. Is the manuscript technically sound, and do the data support the conclusions?

Reviewer #1: Partly

**********

2. Has the statistical analysis been performed appropriately and rigorously?

Reviewer #1: No

**********

3. Have the authors made all data underlying the findings in their manuscript fully available?

The PLOS Data policy

Reviewer #1: No

**********

4. Is the manuscript presented in an intelligible fashion and written in standard English?

Reviewer #1: Yes

**********

Reviewer #1: This study identifies an exploratory four-gene mucosal transcriptomic signature associated with vedolizumab response in ulcerative colitis and further examines its transferability to peripheral blood. The topic is of potential interest; however, several methodological and interpretative issues should be addressed to better support the conclusions.

1.The rationale for avoiding a formal cross-platform validation is understandable. However, it remains unclear why an exploratory sensitivity analysis in E-MTAB-7845 was not performed. Such an analysis, if clearly labeled as exploratory, could still provide useful information regarding the reproducibility of the mucosal signal.

2.The conclusion that the signal is “tissue-restricted” appears too strong. The mucosal and blood cohorts differ not only in tissue type, but also in platform, cohort characteristics, and response definitions. Therefore, the poor performance in blood cannot be attributed specifically to tissue compartment.

3.The formatting of statistical notation and tables should be standardized. In particular, P values should be italicized throughout the manuscript, and table formatting should be revised for greater consistency and clarity.

4.The interpretation of the interferon-gamma finding appears somewhat overstated. The observed difference did not reach conventional statistical significance (P = 0.069), and therefore this result should be more clearly framed as exploratory rather than as strong biological or mechanistic evidence.

**********

what does this mean?). If published, this will include your full peer review and any attached files.

If you choose “no”, your identity will remain anonymous but your review may still be made public.

**Do you want your identity to be public for this peer review?**  For information about this choice, including consent withdrawal, please see our Privacy Policy

Reviewer #1: No

**********

[NOTE: If reviewer comments were submitted as an attachment file, they will be attached to this email and accessible via the submission site. Please log into your account, locate the manuscript record, and check for the action link "View Attachments". If this link does not appear, there are no attachment files.]

To ensure your figures meet our technical requirements, please review our figure guidelines: https://journals.plos.org/plosone/s/figures

You may also use PLOS’s free figure tool, NAAS, to help you prepare publication quality figures: https://journals.plos.org/plosone/s/figures#loc-tools-for-figure-preparation.

NAAS will assess whether your figures meet our technical requirements by comparing each figure against our figure specifications.

# Author comment: Author response to Decision Letter 1

**DOI:** 10.1371/journal.pone.0361301.r002

26 Sep 2026

We thank the Academic Editor and the reviewer for their constructive comments. Our detailed point-by-point responses are provided in the uploaded “Response to Reviewers” document. We have also uploaded the revised manuscript and a version with tracked changes showing the revisions relative to the original submission.

Attachment Submitted filename: Response_to_Reviewers.docx

# Peer review: Decision Letter 1

**DOI:** 10.1371/journal.pone.0361301.r003

**Authors:** Lanlan Chen

9 Oct 2026

Exploratory baseline mucosal transcriptomics and vedolizumab response in ulcerative colitis

PONE-D-26-17559R1

Dear Dr. Wang,

We’re pleased to inform you that your manuscript has been judged scientifically suitable for publication and will be formally accepted for publication once it meets all outstanding technical requirements.

Within one week, you’ll receive an e-mail detailing the required amendments. When these have been addressed, you’ll receive a formal acceptance letter and your manuscript will be scheduled for publication.

An invoice will be generated when your article is formally accepted. Please note, if your institution has a publishing partnership with PLOS and your article meets the relevant criteria, all or part of your publication costs will be covered. Please make sure your user information is up-to-date by logging into Editorial Manager at Editorial Manager® and clicking the ‘Update My Information' link at the top of the page. For questions related to billing, please contact billing support.

If your institution or institutions have a press office, please notify them about your upcoming paper to help maximize its impact. If they’ll be preparing press materials, please inform our press team as soon as possible -- no later than 48 hours after receiving the formal acceptance. Your manuscript will remain under strict press embargo until 2 pm Eastern Time on the date of publication. For more information, please contact onepress@plos.org.

Kind regards,

Lanlan Chen

Academic Editor

PLOS One

Additional Editor Comments (optional):

Reviewers' comments:

Reviewer's Responses to Questions

**Comments to the Author**

Reviewer #1: All comments have been addressed

**********

2. Is the manuscript technically sound, and do the data support the conclusions?

Reviewer #1: Yes

**********

3. Has the statistical analysis been performed appropriately and rigorously?

Reviewer #1: Yes

**********

4. Have the authors made all data underlying the findings in their manuscript fully available?

The PLOS Data policy

Reviewer #1: Yes

**********

5. Is the manuscript presented in an intelligible fashion and written in standard English?

Reviewer #1: Yes

**********

Reviewer #1: The authors have carefully revised the manuscript and adequately addressed the concerns raised during the previous round of review.

The revisions have substantially improved the transparency, rigor, and clarity of the manuscript. I have no further concerns and recommend acceptance for publication in PLOS ONE.

**********

what does this mean?). If published, this will include your full peer review and any attached files.

If you choose “no”, your identity will remain anonymous but your review may still be made public.

**Do you want your identity to be public for this peer review?**  For information about this choice, including consent withdrawal, please see our Privacy Policy

Reviewer #1: No

**********

# Editor report: Acceptance letter

**DOI:** 10.1371/journal.pone.0361301.r004

**Authors:** Lanlan Chen

PONE-D-26-17559R1

PLOS One

Dear Dr. Wang,

I'm pleased to inform you that your manuscript has been deemed suitable for publication in PLOS One. Congratulations! Your manuscript is now being handed over to our production team.

At this stage, our production department will prepare your paper for publication. This includes ensuring the following:

* All references, tables, and figures are properly cited

* All relevant supporting information is included in the manuscript submission,

* There are no issues that prevent the paper from being properly typeset

You will receive further instructions from the production team, including instructions on how to review your proof when it is ready. Please keep in mind that we are working through a large volume of accepted articles, so please give us a few days to review your paper and let you know the next and final steps.

Lastly, if your institution or institutions have a press office, please let them know about your upcoming paper now to help maximize its impact. If they'll be preparing press materials, please inform our press team within the next 48 hours. Your manuscript will remain under strict press embargo until 2 pm Eastern Time on the date of publication. For more information, please contact onepress@plos.org.

You will receive an invoice from PLOS for your publication fee after your manuscript has reached the completed accept phase. If you receive an email requesting payment before acceptance or for any other service, this may be a phishing scheme. Learn how to identify phishing emails and protect your accounts at https://explore.plos.org/phishing.

If we can help with anything else, please email us at customercare@plos.org.

Thank you for submitting your work to PLOS One and supporting open access.

Kind regards,

PLOS One Editorial Office Staff

on behalf of

M.D Lanlan Chen

Academic Editor

PLOS One
